# Activation of a Secondary‐Messenger Receptor via Allosteric Modulation of a Dynamic Conformational Ensemble

**DOI:** 10.1002/anie.202509394

**Published:** 2025-08-06

**Authors:** Benedikt Söldner, Himanshu Singh, Elias Akoury, Gregor Witte, Rasmus Linser

**Affiliations:** ^1^ Department of Chemistry and Chemical Biology TU Dortmund University Otto‐Hahn‐Str. 4a Dortmund 44227 Germany; ^2^ Department of Chemistry and Pharmacy Ludwig‐Maximilians University Butenandtstrasse 5–13 Munich 81377 Germany; ^3^ Department of Biosciences and Bioengineering Indian Institute of Technology Guwahati Guwahati Assam 781039 India; ^4^ Department of Physical Sciences Lebanese American University Beirut 11022801 Lebanon; ^5^ Gene Center Ludwig‐Maximilians University Feodor‐Lynen Strasse 25 Munich 81377 Germany

**Keywords:** Conformational exchange, Cyclic‐di‐AMP, Nuclear magnetic resonance spectroscopy, Protein dynamics, Secondary messengers

## Abstract

Bacterial signaling cascades have recently become of great relevance in the context of bacterial antibiotics resistance. Cyclic diadenylate monophosphate (c‐di‐AMP) is a key bacterial secondary messenger involved in growth, biofilm formation, virulence gene expression and others. The activation mechanisms of c‐di‐AMP receptors like the trimeric P_II_‐like proteins upon messenger binding have, however, remained elusive due the pivotal role of highly flexible protein regions. Here, using solution NMR spectroscopy to elucidate the interplay between the ordered and disordered structural elements of the apo and messenger‐bound forms of the 44 kDa homotrimeric P_II_‐like signal transduction protein A (PstA), we reveal a sensitive modulation of the conformational ensemble of those extended loops thought to bind the downstream interaction partners by messenger association at the receptor core. The orchestration of the spatial properties of the loops, despite their retained internal dynamics, reveals the importance of allosteric effects even for disordered structural elements, whose steerable ensemble properties have long escaped the classical structural‐biology understanding.

## Graphical abstract

Intrinsically disordered loops within globular protein domains have long escaped structural biology. Here, Söldner et al. show that despite this disorder, an allosteric modulation is exerted in a secondary‐messenger receptor by binding of its secondary messenger. This effectively reorients the conformational ensemble of its binding interfaces without abrogating their high degree of dynamics.

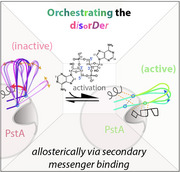

## Introduction

The ubiquitous role of intrinsically disordered proteins/regions (IDPs/IDRs) for protein function in almost any context of cellular organization and activity has widely been recognized in the last decade, owing to a soaring number of experimental studies and simulations.^[^ ^1^, ^2^, ^3^, ^4^ ^]^ Their diverse functional aspects, long elusive due to the lack of any defined conformations, can be rationalized today by an ensemble description,^[^ ^5^, ^6^, ^7^ ^]^ which quantitatively rationalizes their selectivities and overall functionalities derived from partially preformed transient structures, folding upon binding, or mixtures of both.^[^ ^8^, ^9^, ^10^ ^]^ Despite the rapid interchange of conformations, the properties of these ensembles are well‐defined, context‐specifically tailored, and evolutionarily fine‐tuned. While retaining a high degree of plasticity, these still define important biophysical features, for example, condensation tendencies or pore permeabilities via distinct steric and electrostatic effects.^[^ ^11^, ^12^, ^13^ ^]^

Loop structures within folded domains, beyond their obvious role for interconnecting the classical secondary‐structure elements, would be expected to constitute equally important elements for tailored affinity, cooperativity, and propagation of information across signaling cascades. However, how events within the flexible loops can possibly impact functional features of the protein core or, vice versa, the possible dependency of loop properties on events in the core, has largely escaped the current structural‐biology understanding and requires careful analysis of interdependencies in the dynamic ensemble.

**Figure 1:**
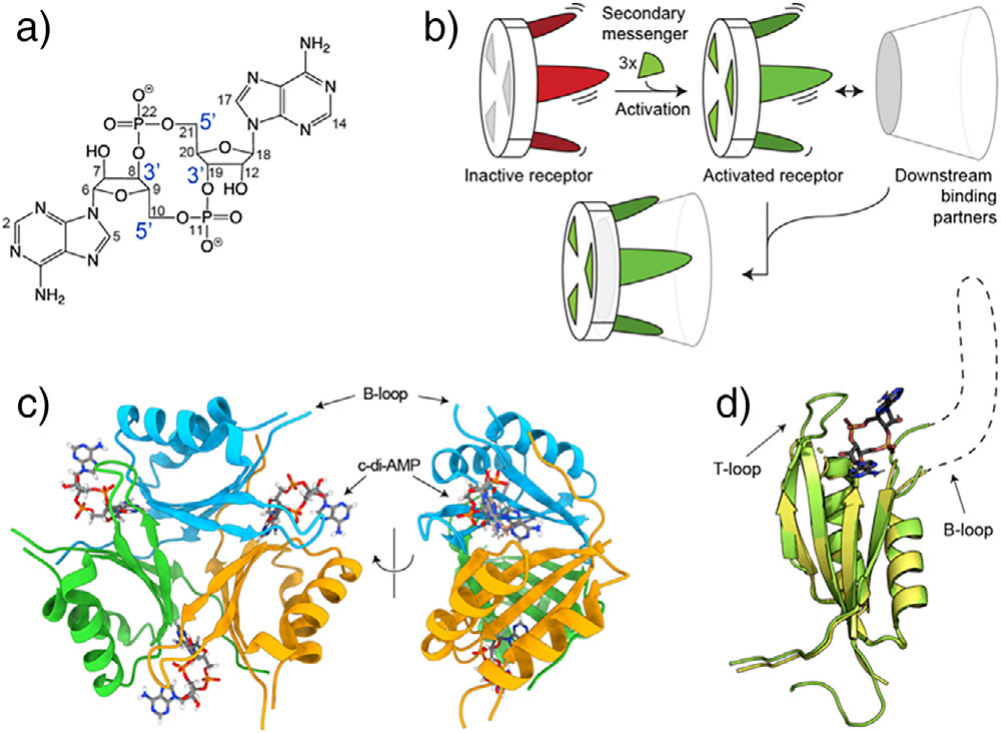
PstA structure and secondary‐messenger binding. a) Cyclic di‐AMP (c‐di‐AMP), a bacterial secondary messenger. b) Current model of activation of P_II_‐like secondary‐messenger receptors by c‐di‐AMP binding. A binding‐incompetent trimeric receptor becomes activated by threefold association of c‐di‐AMP, allowing its long, disordered loops to coordinate *C*_3_‐symmetric downstream interaction partners.^[^ ^16^ ^]^ c) Trimeric arrangement and structural elements of PstA represented by the X‐ray coordinates of the holo complex (PDB 4WK1). d) Overlay of crystal structures of apo‐PstA (PDB 4WK3) and (c‐di‐AMP)_3_:(PstA)_3_ complex (PDB 4WK1), depicting a single monomer.

Here, we interrogate this cross‐coupling in a bacterial secondary‐messenger receptor, where messenger association happens to the core, but binding to downstream interaction partners hinges on long, disordered loops, whose structural properties have escaped structural biology and, on the first glance, seem fully unrelated to messenger binding in the core of the trimeric complex.^[^ ^14^ ^]^ Secondary messengers are intracellular messengers that act in response to an extracellular signal, which are generated at cell surface receptors to initiate downstream processes within the cell while prohibiting the primary messenger from overcoming the cell membrane.^[^ ^15^ ^]^ Bacterial secondary‐messenger systems, many of which are non‐existent in humans, have seen tremendous scientific interest in recent years in particular due to the rise of bacterial drug resistance, a soaring problem with a large societal impact, as they provide a means to selectively combat procaryotes. Cyclic diadenylate monophosphate (c‐di‐AMP, see Figure 1a), catalyzed by diadenylyl cyclase (DAC) enzymes through the condensation reaction of two ATP molecules,^[^ ^17^ ^]^ is an important secondary messenger widely found in prokaryotes, regulating sensing of DNA integrity, cell wall metabolism, and potassium transport.^[^ ^17^, ^18^, ^19^ ^]^ Despite intense efforts in identifying the mechanisms of cyclic dinucleotides with their protein binding partners for therapeutic intervention, however, the activation of the respective receptors by c‐di‐AMP secondary messenger binding are poorly understood.

Commonly found among Gram‐positive firmicutes and actinobacteria,^[^ ^18^ ^]^ c‐di‐AMP binds several receptors including the transcription factor BusR, the cytoplasmic cation transporters KtrA and CpaA, and the histidine kinase KdpD.^[^ ^20^, ^21^ ^]^ The family of P_II_ signal transduction proteins is among the most widespread in bacteria, archaea, and plants. Upon contact with the vital effector molecules ATP/AMP and 2‐oxoglutarate, it signals on nitrogen availability and interacts with other enzymes, transcription factors, and membrane transport proteins.^[^ ^22^, ^23^, ^24^ ^]^ Trimeric P_II_ proteins are known to bind trimeric target proteins like amphotericin B (AmtB), N‐acetylglucosamine kinase (NAGK), PipX protein, and dinitrogenase reductase‐activating glycohydrolase (DraG).^[^ ^25^ ^]^ They are highly conserved homotrimers (of around 40 kDa overall molecular weight) exhibiting a double βαβ ferredoxin‐like fold (compare Figure S1).^[^ ^26^, ^27^ ^]^ Each monomer comprises a compact core of two α‐helices and four β strands prominently connected by two loops that accommodate intermolecular ligand and ATP‐binding sites.^[^ ^28^ ^]^ With an arrangement of three long protruding loops (called either B‐loop or T‐loop), one from each of the monomers, the trimer is able to capture downstream interaction partners (compare Figure S2, PDB 2nuu^[^ ^16^ ^]^), thereby activating the associated signaling cascades (scheme in Figure 1b). The activation mechanism of these receptors has, however, remained elusive as the disordered loops have largely been invisible in crystal structures. In all those cases in which a reasonably defined electron density for part of the loops was obtained (compare Figure S1), their observed structural features likely originate from the intermolecular crystal contacts and must be treated with care. P_II_‐like signal transduction protein A (PstA, Figure 1c) from *Staphylococcus aureus* (named DarA in *B. subtilis*) is one of the various c‐di‐AMP receptors of this fold.^[^ ^20^, ^29^ ^]^ Ligand incorporation in P_II_ proteins generally is thought to be cooperative, meaning that association of the second and third ligand happens with altered affinity to be able to sample a wider range of ligand concentrations.^[^ ^30^, ^31^ ^]^ Messenger binding in PstA happens with a dissociation constant *K*_D_ of around 20 nM (see ITC data in Figure S4 and compare Müller et al.^[^ ^14^ ^]^). The mode of activation by c‐di‐AMP has remained elusive hitherto.^[^ ^29^, ^32^, ^33^ ^]^ Upon ligand binding, the short T‐loop now produces a well‐defined electron density, but the core retains a virtually identical structure with or without the ligand (Figure 1d). The longer B‐loops on the other hand, invisible in both, apo and bound forms, remain fully disordered, posing the puzzle how messenger binding would induce their binding competence. Whereas the role of disordered regions often escapes X‐ray crystallography or cryo electron microscopy, NMR spectroscopy in solution^[^ ^34^, ^35^, ^36^, ^37^, ^38^, ^39^, ^40^ ^]^ or in the solid state^[^ ^41^, ^42^, ^43^, ^44^ ^]^ can assess both, well‐structured regions as well as those that bear no or little (transient) structural properties, facilitating insights into the well‐tempered interplay of structure and dynamics for biological functionality. Here, we use NMR spectroscopy in solution, supported by simulations, of the dynamic ensemble to unveil an intimate coupling between the structural features of the disordered loops and those of the core as a function of secondary‐messenger binding.

## Results and Discussion

### Residual Secondary‐Structural Propensities of PstA Loops

**Figure 2:**
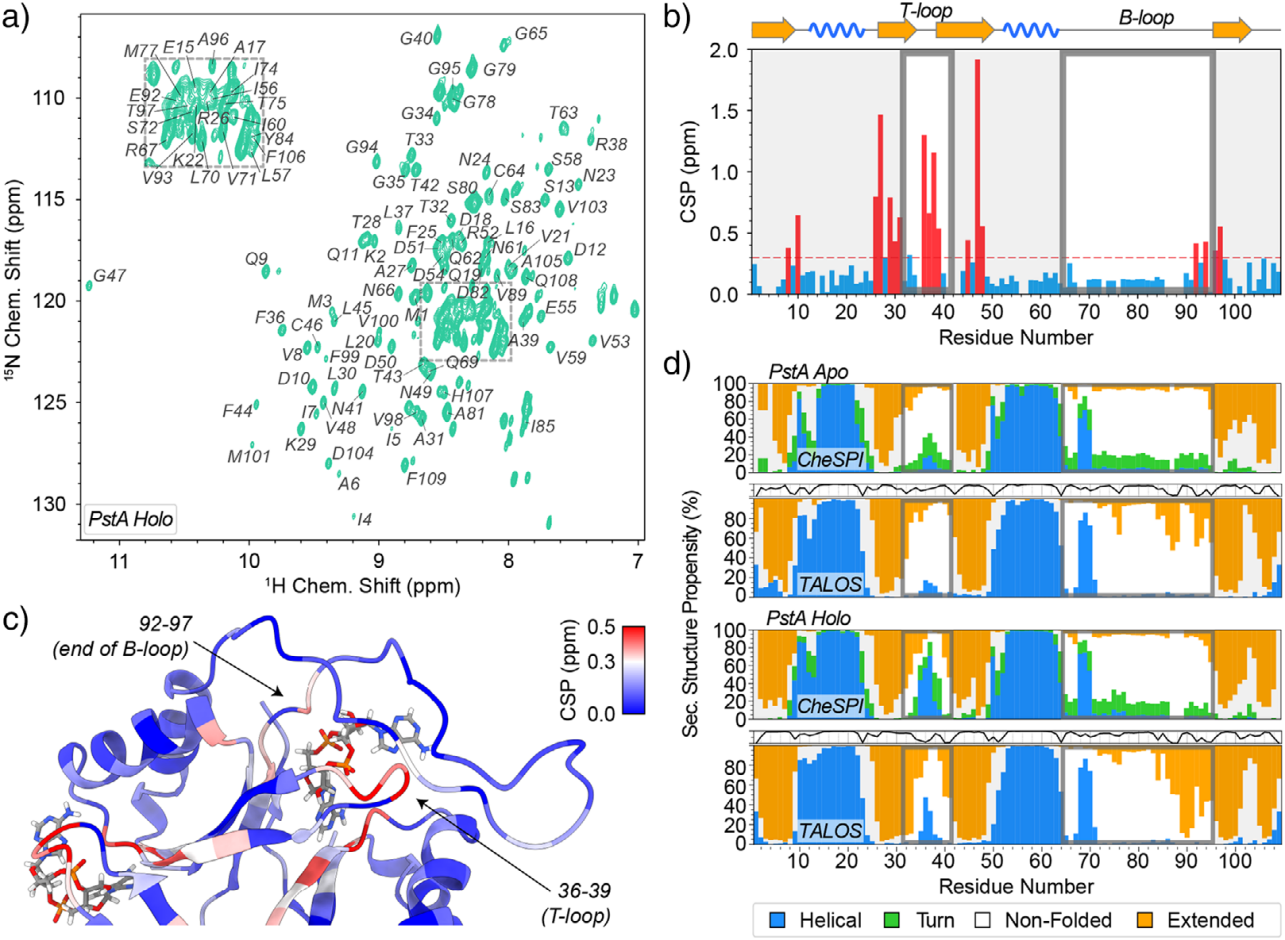
Chemical‐shift perturbations (CSPs) between apo and c‐di‐AMP‐bound PstA and NMR‐based secondary‐structural features. a) 2D 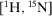
‐HSQC NMR spectrum of the perdeuterated, 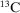/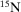
‐labeled c‐di‐AMP‐PstA complex, recorded at 800 MHz Larmor frequency at 25 °C, together with residue‐specific H/N assignments. b) CSPs as a function of residue and c) plotted on the structure. The red line in b) was arbitrarily set to 0.3 ppm. The color code for c) is depicted in the top right. CSPs based on amide proton and amide nitrogen chemical shifts as $\sqrt{\Delta \delta_{H}^{2}+{\left(0.14\cdot \Delta \delta_{N}\right)}^{2}}$. d) Secondary‐structural propensities of apo protein (top) and complex (bottom), assessed either via CheSPI or via TALOS‐N, the loops in focus being highlighted by gray boxes. The TALOS‐N profiles also denote the certainty with which the residue‐specific prediction is associated on top of the respective plot, with a confidence scale ranging from 0 (bottom) to 100 (top).

To assess structural and dynamical features of the PstA receptor and their modulation by secondary‐messenger binding experimentally, we subjected the trimeric complex in the presence and absence of the ligand c‐di‐AMP to solution NMR spectroscopy. Sample preparation was done as described for crystallography previously, however, using either double‐ (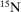/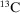) or triple‐labeled (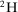/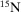/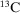) minimal media. (See details on sample preparation in the Materials and Methods). Resonance assignment was achieved using a set of standard triple‐resonance backbone experiments. (See details in the Materials and Methods. Figure S5 shows an exemplary sequence of strips obtained from an HNCACB experiment.) In total 504/437 shifts (CO, C^α^, C^β^, N^H^, and H^N^) from 106/98 amino acids (95.8%/83.1% of all CO, C^α^, C^β^, N^H^, and H^N^ shifts; 96.2%/85.6% of all backbone amide shifts) could be unambiguously assigned for ligand‐bound and ligand‐free PstA, respectively. (Figure 2a shows a 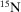
‐HSQC spectrum of the receptor in c‐di‐AMP‐bound form. All resonance assignments were deposited in the BMRB under accession codes 52 882 and 52 883.) Upon binding of the secondary messenger, large chemical‐shift differences (CSPs) are observed in the T‐loop and in the end of β3, that is, sites that are in direct contact with the ligand and particularly through interactions involving G47, F36, L37, and R26. (See an overlay of the 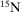
‐HSQC spectra, residues with CSPs >1.0 ppm being highlighted in Figure S6, as well as chemical‐shift perturbations plotted on the monomeric protein structure and as a function of residue in Figures 2b, c, respectively. Figure S7 shows a 3D HSQC‐NOESY strip highlighting G47 to ligand cross peaks.) Interestingly, in addition, residues 92 to 97, the C‐terminal residues of the B‐loop, show sizable perturbations after addition of the ligand.

Secondary‐structural features of the trimer core, assessed using either CheSPI^[^ ^45^ ^]^ or TALOS‐N^[^ ^46^ ^]^ (see Figure 2b), exactly match the observations from previous crystallographic studies. The T‐loop, compared to the apo form, attains a substantial shift from non‐folded toward structured behavior. Interestingly, however, apart from a slight increase of extended propensity at the very end of the B‐loop, which is in relative proximity to the ligand, and a slightly more helical tendency for residues 75–77, the very beginning of the B‐loop, the highly dynamic properties of the B‐loops seem to be largely maintained upon binding of the messenger. Only the *residual*, transient structural propensities in these regions seem to be influenced by messenger binding, while their high degree of disorder is very clear throughout residues 73–96. A rather steady helical stretch, irrespective of messenger binding, is seen for residues 68–70, even though these residues are disordered in crystallography.

### Conformational Dynamics of Apo and Ligand‐Bound Forms of PstA

**Figure 3:**
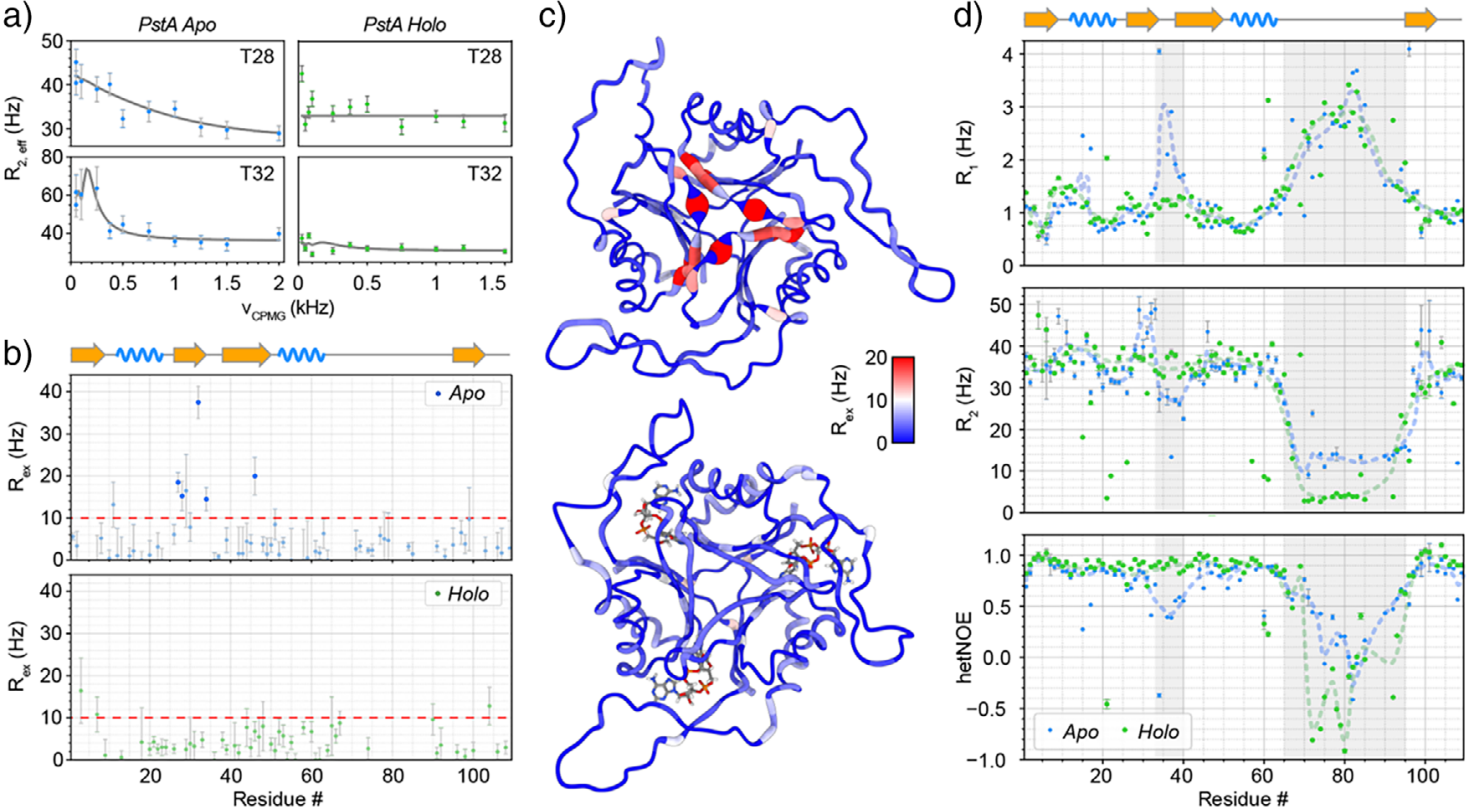
Conformational dynamics in apo and messenger‐bound PstA. a) Representative relaxation dispersion profiles for apo PstA (left), compared to those of the same residues of the complex (right). b) Exchange contributions *R*_ex_ as a function of residue, comparing complex (bottom) and apo protein (top). The points clearly above the red line reflect strong dispersion as opposed to noise‐like fluctuations. c) *R*_ex_ contributions plotted on the structure, comparing apo protein (top) and complex (bottom). d) Longitudinal relaxation rates *R*_1_, transverse relaxation rates *R*_2_, and heteronuclear steady‐state NOE for apo protein (blue) and complex (green). Gray shades denote residues in the T‐loop (residues 33–40) and B‐loop (residues 65–95). Dashed trend lines disregard local outliers found for individual residues such as in short turns.

To assess, in more quantitative detail, the dynamics of individual amino acids in the complexes, we characterized motion on different timescales, the intermediate (µs‐ms) and fast‐timescale (ps‐ns) dynamics, both represented in Figure 3. To characterize the site‐specific µs‐ms conformational‐exchange dynamics of the PstA trimer, we engaged 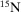 constant‐time CPMG relaxation dispersion experiments (see Experimental Details in the SI). Interestingly, for the apo trimer, strong relaxation dispersion (with an exchange contribution of up to 38 s^−1^), denoting slow µs‐timescale motion on the 100 µs timescale (see Table S1), is found for the majority of residues in β2, one residue in the beginning of the T‐loop, and in one β3‐residue (see Figure 3a–c). Most of these residues span the trimeric interface, which is formed predominantly by the residues shortly before the T‐loop (residues 27–34). Exchange contributions *R*_ex_ from individual fits are depicted as a function of sequence in Figure 3b and color‐encoded on the structure in Figure 3c. This presence of relaxation dispersion demonstrates a conformational exchange in the core of the apo form. By contrast, this exchange is fully abolished upon ligand binding, where no dispersion is witnessed over the entire protein (Figure 3a–c). Given the large size of the trim (and hence limitations regarding the signal‐to‐noise ratio), the data do not allow an accurate determination of the timescale, but the presence/absence of strong conformational exchange is unambiguous. Given the colocalization of residues with an exchange contribution and the intermolecular interactions, the observations likely point to a temporary loosening of the trimer interface, which might serve a facilitated uptake of the bulky ligand. In the bound form, by contrast, the intermolecular interactions are significantly strengthened through multiple additional indirect intermolecular contacts mediated by the ligand (Figure S8), hence rigidifying not only the T‐loop, expected from crystallography and described below, but also abrogating conformational exchange of the trimer interface. Interestingly, the CPMG experiments do not show a significant extent of dispersion for the residues of the B‐loop. A contribution to the *R*_2_ rates from conformational exchange here (which are different between the apo and holo forms, see below) must hence derive from intermediate‐timescale processes faster than accessible via relaxation dispersion methods, that is, beyond the ∼50 µs timescale. In addition, fast‐timescale (ps‐ns) dynamics for apo and ligand‐bound PstA were obtained using 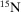 *R*_1_, *R*_2_, and heteronuclear NOE experiments (Figure 3d, top to bottom, respectively). In the apo form, the T‐loop is characterized by hetNOE values much lower (down to ∼0.5) and *R*_1_ values much higher (up to 4 s^−1^) than the prototypical core residues locally, suggesting high flexibility on the ps‐ns timescale. By contrast, in the presence of c‐di‐AMP, the low hetNOE and high *R*_1_ values of the apo form are completely flattened out, all parameters now matching those of the other core residues. This suggests a complete rigidification of the T‐loop in the bound form due to the interactions with the ligand. Ligand binding, involving a total of 8 H‐bonds to the receptor (compare Figure S8) hence locks the T‐loop into a single, well‐defined position. In the apo form, just before and after the T‐loop close to the ligand binding site, very high *R*_2_ values up to ∼50 s^−1^ are found, which is in congruency with the relaxation dispersion data above. These rates exceed the values of around 35 s^−1^ found throughout the remainder of core residues (which are in congruency with what would be expected for a perdeuterated ∼40 kDa globular protein at 30 °C), confirming the presence of the intermediate‐timescale conformational exchange process described above.

In coherence with their disordered character in crystallographic studies, most of the B‐loop residues (including amino acids 69–93) show the signatures of fast‐time scale mobility. In contrast to the well‐defined density for the end and beginning of the loop in part of the X‐ray structures, the NMR data suggest steadily increasing disorder toward the center of the loop in both, the apo and holo form. Residues at the very center of the B‐loop have similarly high *R*_1_ values for both forms, and also hetNOE values confirm the high degree of local motion for both forms, with slightly less extreme tip dynamics for apo than for holo PstA. Maybe most interestingly, differences are observable with respect to the *R*_2_ rates of the loop center: Whereas the holo form yields values of around 2–4 s^−1^, such rates in the B‐loop tip (but not for the rest of the protein) are ∼4 × higher (by around 10 s^−1^) in the apo form. Given the hetNOE and *R*_1_ data, fast‐timescale dynamics with very low effective correlation times still clearly apply for both forms. This can be rationalized intuitively by dissecting the two individual underlying contributions (timescales) to *R*_2_, where the higher *R*_2_ rates in the apo loops likely point to differential chemical‐exchange (*R*_ex_) contributions. Without any surface interactions, apo and holo loops would bear identical contributions from fast, internal ps – ns timescale reorientation of the individual H─N bond vectors. However, an extra contribution from (µs timescale) chemical exchange (varying fast‐timescale averages) due to short‐lived but repeated interactions with the structurally variable ligand binding site, modulating the chemical shifts on a slower timescale and hence adding to dephasing, will depend on the presence/absence of the c‐di‐AMP ligand just below it, while retaining the fast‐motional profiles regarding *R*_1_ and hetNOE. By contrast, such conformational‐exchange contributions seem different (lower) for the holo loop tip, suggesting that the ligand somehow drives the center of the loop into a more homogeneous situation with fewer surface contacts, dominated by the fast internal motion. Even though these are slight differences between apo and holo trimers, the data are compatible neither with defined loop conformations nor with loops that, irrespective of messenger binding, move fully independently. Instead, the NMR experiments hint to alterations of transient interactions between dynamic loops and the receptor core upon messenger binding, hence somehow modulating the spatial properties of the loop and redefining interaction sites available in the ensemble for downstream signaling.

### Simulation of PstA Dynamics

In order to complement the differences in loop behavior witnessed experimentally in the conformational ensemble in the holo form compared to the apo receptor with more mechanistic details, we pursued simulations both, for the apo as well as for the ligand‐bound protein. (See the SI for building of ligand topology and parametrization as well as further details on the setup of these simulations.) Gaussian‐accelerated (GaMD) simulations with a high boost potential (See details in the SI.) were used to obtain six complex structures of three monomers each that sufficiently cover the available conformational space. These starting structures (in total 18 loop conformations under otherwise identical conditions) hence obtained were used to start brute‐force MD replica simulations (yielding in total 18 µs of simulation time with respect to the conformational space sampled by the loops). This way, the bias associated with a single starting structure, the danger of being trapped in local energy wells, as well as the potential pitfalls associated with reconstructing motional properties from accelerated simulations were circumvented. Figure S9 shows root‐mean‐square deviation (RMSD) with respect to the respective starting structure as a function of time. As during the first 100 ns most RMSDs reach a plateau, all further analyses were performed based on the remaining 900 ns, also for reducing even further a potential bias by the starting structure.

**Figure 4:**
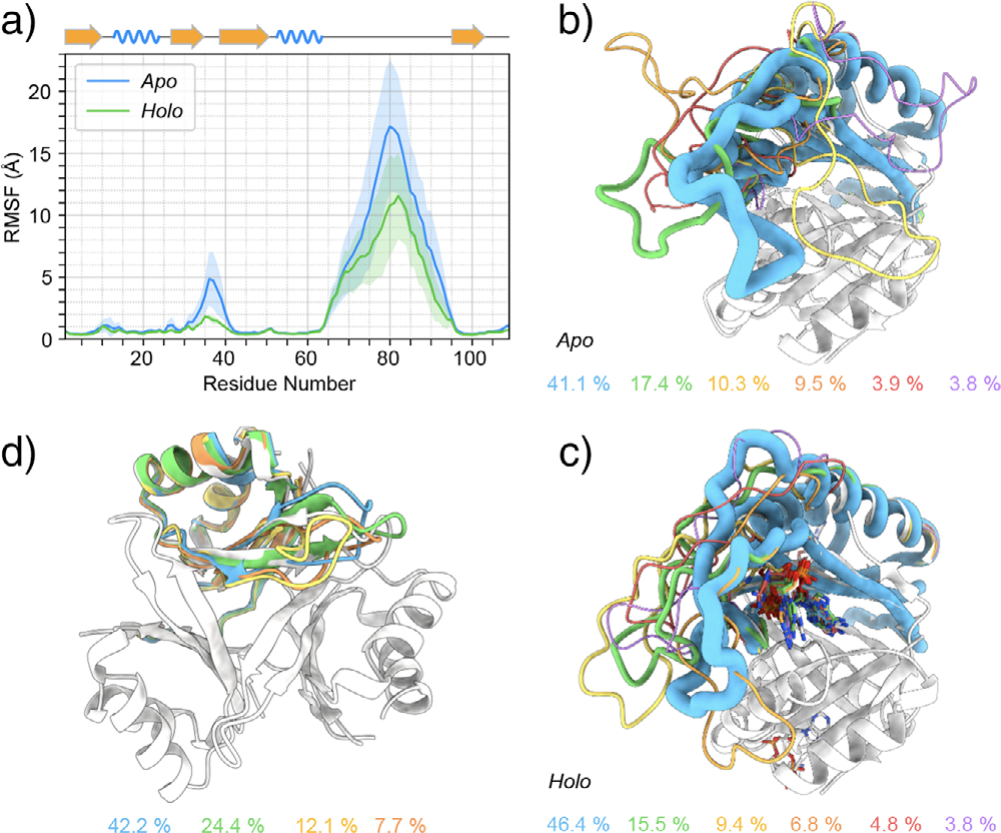
Conformational space sampled by the PstA core and B‐loop as obtained by simulations. a) Root mean square fluctuations with respect to the average structure of both trajectories as a function of residue, comparing the apo complex (blue) with the messenger‐bound form (green). b,c) Cluster analysis of the conformational ensemble of the apo (b) and holo receptor (c), using RMSD cutoffs of 5.0 and 3.5 Å, respectively. For cluster analysis, a concatenated trajectory of all three single monomers was used. d) Conformational distribution of the apo form core and T‐loop only, assessed by clustering with an RMSD cutoff of 1.2 Å. Whereas the behavior of the tip of the T‐loop matches the fast‐timescale motion seen experimentally, structural changes of the interface, as expected on the 100 µs timescale from dispersion measurements, are not captured within the much shorter simulation. A peptide flip is observed for residue 27 – 28, compare Figures S12 and S13. (The conformations of the holo form are captured within a single such cluster, which is hence not shown.) For b–d), the relative populations of each cluster are denoted in a descending order below.

As expected, the fast overall motion of the B‐loop observed experimentally is well‐reflected in both forms. However, extended structure is observed as temporary events that form sporadically in the beginning and the end of the B‐loop in holo form. (See residue‐specific secondary‐structural propensities as a function of time in Figure S10.) Interestingly, the Cartesian space covered by overall loop motion is significantly higher in the apo form compared to the holo trimer. Figure 4a shows this deviation (root‐mean‐square fluctuations, RMSFs) in Cartesian space as a function of residue, where higher values for both, the T‐ and the B‐loop, are obtained in the apo form. To elucidate the nature of these conformational dynamics in more detail, the trajectories were clustered based on backbone atoms (N, C^α^, C^β^, C°, N, and H^N^) with the GROMOS algorithm described by Daura et al.^[^ ^47^ ^]^ The cutoff values for clustering the conformational space of the B‐loop using concatenated monomer trajectories was set such that 85 % of the conformational space could be captured within six clusters, that is, a 5.0 Å cutoff for the more divergent apo form and a 3.5 Å cutoff for the less divergent holo form. Figure S11 demonstrates that transitions between the clusters frequently occur. Figure 4b, c visualize the clusters obtained for apo and holo form. Whereas the holo form is dominated by a common directionality of the loop, the apo form samples a wide range of directions and surface interactions. The high degree of *local* disorder (i.e., bond vector reorientations) toward the middle of the loop is, however, retained for both forms, as expected from the experimental *R*_1_ and hetNOE values. For analyzing the conformational space sampled by the *core* and the small T‐loop, the coordinates of apo and holo trimers *without* the B‐loop were clustered using a cutoff of 1.3 Å. For the apo form, for this cutoff, 85% of the frames were captured within four clusters (depicted in Figure 4d), whereas for the holo form, a single cluster already covers the entire conformational space sampled. The motions within the monomer‐monomer interface of the core, a conformational exchange experimentally observed on a >100 µs timescale (vide supra), cannot be not expected to be captured in MD and is not seen in the simulation. By contrast, the behavior of the tip of the T‐loop in the apo form matches the fast‐timescale motion seen experimentally.

**Figure 5:**
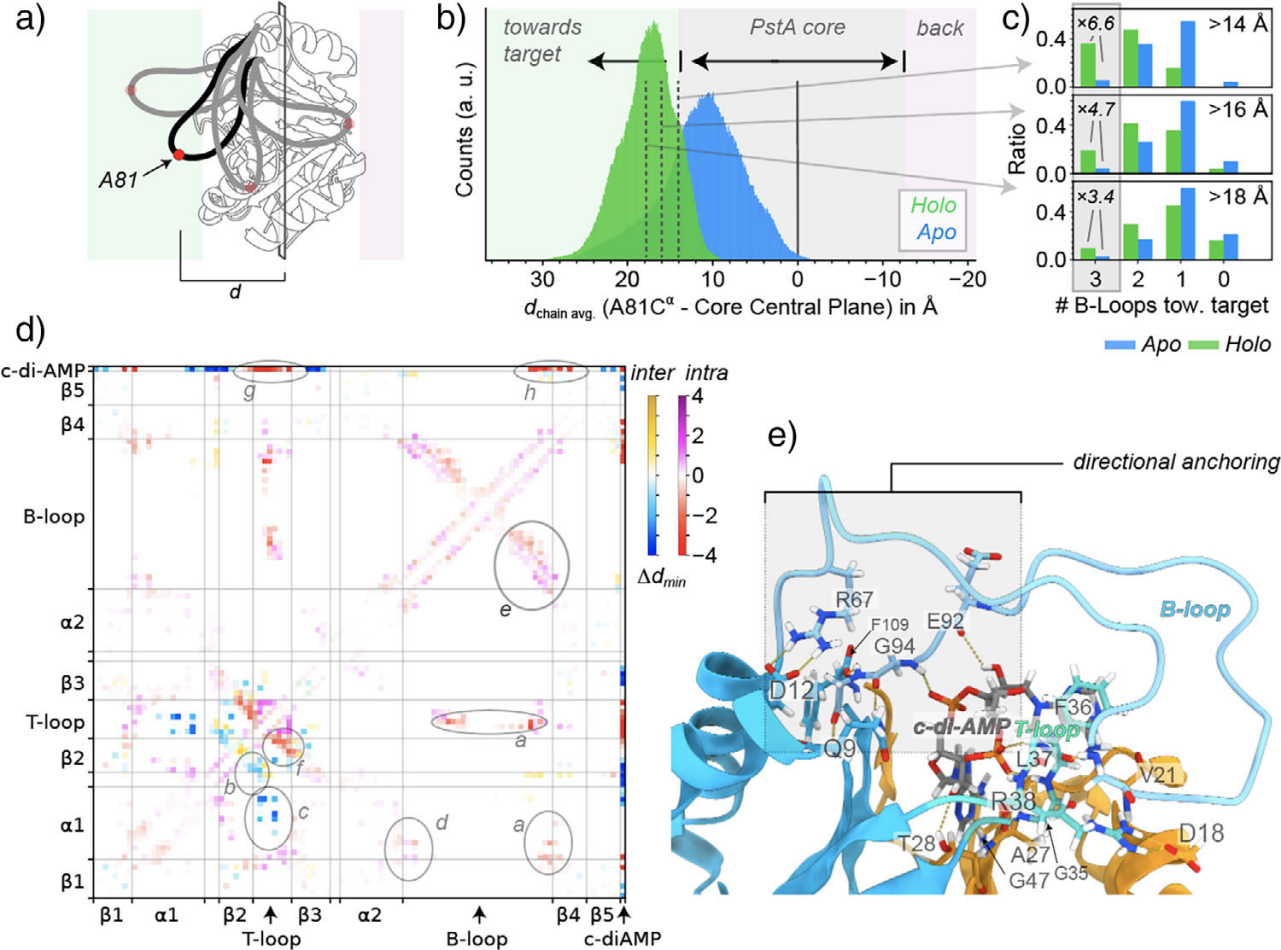
Mechanistic assessment of loop pre‐direction. a) Quantification of loop extrusion via distance measurement between residue 81 at the outer tip of the B‐loop and the central plane through the center of mass of the core and perpendicular to the *C*_3_ axis. b) Distribution of loop protrusion, depicted as average over all three monomers at any given time, measured as depicted in a). c) Distribution of *collective* forward positioning (3, 2, 1, or none of the loops being beyond a given threshold) as a function of the threshold chosen (on the upper right, also compare Figure S16). The numbers on the bottom left denote the ratio of occurrence of three forward‐positioned loops between holo and apo receptor. d) Differences in intra‐ and intermolecular interactions between apo and holo PstA, measured as the difference in minimum distance between residues. (Red and blue show decreased distances for intra‐ and intermonomer contacts and hence closer proximity, compare Figure S17 for the individual minimal distances.) Ligand association entails an increase for contacts between T‐loop and B‐loop *a*), between T‐loop and β2 (intermolecular, *b*), between T‐loop and α1 (intermolecular contacts, *c*), a shift of the contact distribution between the end of the B‐loop and the beginning of α1 (*d*), a shift of the intra‐B‐loop contact distribution *e*) as well as stronger intra‐T‐loop contacts *f*), in addition to direct interactions between loops and ligand (*g*, *h*). e) Important H‐bonds (>10% relative occurrence in the MD simulations), depicted on a representative conformation of holo PstA, including a first monomer (blue, with the B‐loop and the T‐loop represented in light blue and cyan, respectively) and a second monomer (orange), and the c‐di‐AMP ligand (gray). The network of recurring contacts at the end and beginning of the B‐loop is highlighted with a gray box.

To elucidate in more detail in which way loop dynamics are impacted by the presence of the ligand, we interrogated the in silico ensembles in various ways. Most effectively, a plane was constructed from which the distance of any loop residues could be measured. This plane is perpendicular to the *C*_3_ symmetry axis of the trimer and located in the center of mass of the core, such that *large positive* distances measured for the tips of all three loops, according to the current model (Figure 1b), represent a binding‐competent conformation, while low or negative distances for one or more loops denote binding‐incompetent conformations (Figure 5a). Figure 5b shows the distributions obtained for the apo and holo receptor with respect to the loop position (using residue A81 as a reporter, averaged over the three monomers). The width of the distribution in holo form is significantly narrower and a strong, 7 Å shift of the average distance of this distribution toward the putative interaction side is witnessed. The presence of the ligand hence achieves an effective distributional shift of the space in which the loops prevail. Even though only subtle, a small decrease of *R*_g_ is associated with the loops’ directional bias induced by the messenger, which makes the complex slightly more compact (Figure S14). This observation is in qualitative consistence with SAXS data, which confirm the subtle decrease of the diameter in the presence of c‐di‐AMP also from an experimental standpoint (Figure S15, deposited into the SASBDB under accession codes SASDXD5 and SASDXE5 for apo and holo PstA, respectively).

On a first glance, it is hard to imagine that a subtle repositioning could have an impact on binding competency. However, all three loops are thought to be involved upon binding, leading to a *C*_3_‐symmetric complex. Conversely, assuming a conformational‐preselection model, an encounter complex would translate into a binding event with a higher probability in case of a preformed binding‐competent conformation, which kinetic advantage could be particularly important for the low concentrations of the receptor in the cell. To assess the relative probabilities for a proper interface to form (i.e., *all* loops positioned toward the interaction partner) in the apo versus the holo trimer, we interrogated the ensemble by applying a series of different axial threshold values assumed for sufficient forward protrusion. Indeed, the subtle distributional effect for the average B‐loop position as a single event (Figure 5B) translates into a severalfold probability difference when taking the trimeric nature of the interface into account, which hence exerts a strong amplification of the modulating effect of the ligand (Figures 5c, S16). Assuming ∼16 Å as a representative cutoff for minimum forward protrusion, the chance of forming a binding‐competent interface (three loops pointing forward) is ∼5 times higher than in the apo form. A conformational preselection of the ensemble by the trimeric architecture and its avidity would hence have a substantial kinetic effect, while preorientation of the large loops would further facilitate complex formation via reduced entropic costs of binding, enhancing also the thermodynamic feasibility of binding in addition to the kinetic advantage. To better rationalize the grounds of the redirectional effects upon messenger association in particular for the disordered B‐loop, we performed contact analyses within the dynamic ensemble (compare Figures 5d,e, S17, and Table S2). Indeed, various recurring contacts are observed that, as a whole, embody the shift of the conformational distribution observed in experiments and simulation. Among the most prominent transient contacts are the intermolecular hydrogen bonding contacts between the end of the B‐loop (residue E92 and the conserved motif G94‐G95) and hydroxyls and phosphate of the ligand, which are naturally absent in the apo form. These hydrogen bonds are relatively persistent, with a percental occurrence between 26.3% and 49.4% of the time (compare Table S2). Interestingly, upon ligand binding, G94 and G95 at the end of the B‐loop now also form more persistent *intra*molecular hydrogen bonds with Q9 at the beginning of the α_1_‐helix (18.1% vs. 64.0% of the time for apo and holo form, respectively, for the contact 95GlyO‐9GlnN, but also hydrogen bonds to the sidechain of Q9 are now observed). In addition to this “*latch”*, a rather persistent salt bridge between the conserved residues R67 in the beginning of the B‐loop and D12 in the core is found in apo and holo form (56.7% vs. 67.1% of the time, respectively). This contact tends to act as a *hinge* about which the stem of the loop can rotate in apo, whereas in the holo form, with the “latch” closed, this reorientation is largely restricted. Importantly, all of the residues mentioned above are highly conserved (compare Figure S18 and see below), confirming their dedicated role in restraining the direction of the B‐loop. Furthermore, inter‐loop contacts (mostly hydrophobic contacts, but also patterns of transient hydrogen bonds) are found in the holo form, e.g., interactions between G79 and F36 and between M77 and L37). Lastly, upon stabilization of the T‐loop by ligand binding, manifold transient intermolecular contacts between the T‐loop, the ligand, and the end of the helix α_1_ are traceable. These include both, hydrophobic contacts as well as the formation of various hydrogen bonds (see Table S2). The overall network of these transient inter‐ and intramolecular contacts around the ligand binding site and near to the B‐loop, created or at least strongly reshaped by ligand binding, are consistent with the ensemble being shifted into a spatially more occluded situation whenever the messenger is present. (See Figure S19 for a simplified sketch of these interactions and the effect they entail.) Interestingly, part of the temporary H‐bond network observed as transient features in the holo ensemble also match the ones formed permanently in the crystal structure of holo *L. monocytogenes* PstA (Figure S20, also compare again Figure S1). Generally, the simulation results are in full agreement with the experimental data for the beginning and end of the B‐loop, its moderate chemical‐shift perturbations, and a slight increase in helical/extended propensities despite its retained high degree of internal dynamics.

While the identical apo and messenger‐bound structures of various P_II_‐like proteins from crystallography have consistently made it difficult to associate the event of messenger binding with any sort of biophysical impact, our results—representing a slightly qualitative approach but fraternizing a multitude of complementary viewpoints—reveal a series of interesting differences for both, the core and the loops. On one hand, the above data reveal a so‐far elusive plasticity of the *core* of the apo trimer. It was unforeseen in the original crystallographic studies that the bulky c‐di‐AMP ligand with its extended structure would be found to be buried inside a complex with a structure *identical* to the apo protein. The spontaneous conformational exchange witnessed for the inter‐monomer ties now likely facilitates a partial deformation that will allow quick substrate uptake, while ligand uptake would face strong energetic hurdles in case of a fully rigid geometry. These dynamics, which in the apo form lead to the described dispersion effects on the 100 µs timescale, are quenched upon ligand association as multiple added H‐bonds form, hence further stabilizing the trimeric structure of the holo form.

Even more interestingly, the above analyses also show the characteristics of the disordered and previously “invisible” *loops* to be sensitively modulated by ligand association, which would imply kinetic and thermodynamic differences in associating to downstream interaction partners and hence rationalizes the differences of the complex with respect to an initiation of downstream signaling cascades. Given their seemingly random motional characteristics, the biophysical features of loops in general, other than connecting other structural elements, have largely escaped understanding from a structural‐biology viewpoint and hence not received much interest. The above data show that even with this high degree of local flexibility, overall loop behavior—and hence any functional properties that the loops contribute to—can still be orchestrated from the distance by binding events in the protein core. The B‐loop conformational ensemble is inherently influenced by ligand association, which induces a switch from a rather random loop behavior, stochastically sampling a heterogeneous distribution of transient intramolecular interactions around the ligand binding site, to an effectively directed behavior, where the “stem” of the loop (the C‐ and N‐terminal ends) is rather fixated by fast recurring interactions to the newly shaped and chemically very different ligand binding site, and the tip of the loop instead becomes less involved. The allosteric modulation is noteworthy as the individual amino acids in the loop invariably stay highly flexible with or without the messenger being bound and the regulatory effect solely occurs via a network of concatenated, transient interactions to a redesigned pocket interaction surface. Interestingly, the loop redirection model is also in full congruency with the cooperative character of messenger association assumed for P_II_ proteins earlier, which seems to be an enabling feature for activation of such trimeric receptors. As the properties of the conformational ensemble are a direct consequence of the presence or absence of the messenger, the facilitation of fully activated (triple‐liganded) receptors, favored even at low ligand concentrations, directly translates into predominant formation of a fully binding‐competent interface with respect to the three loops.

To verify our interpretations about the directional role of the loop stem residues, we interrogated the extent of residue type conservation as a function of primary structure over different PstA homologues, as observed in multiple‐sequence‐alignment analysis (Figure S18). Indeed, we obtain perfect agreement with the above model. A highly conserved amino acid motif is identified between PstA residues T28 and T43, with the consensus sequence ^28^TKLXXXGGFLXXGNTT.^[^ ^43^ ^]^ This represents the conserved binding interface to the secondary messenger. More interestingly, amino acids K2, D12, R67, and the amino acid stretch ^94^GGA^96^ toward the C‐terminal end of the protein (compare the contacts described above) are highly conserved and occur in more than 95% of the P_II_‐like protein sequences. While, importantly, these do not bind the ligand, their conservation is congruent with the above architecture of transient loop:core contacts that modulate the B‐loop directional properties as a function of ligand binding. The conserved interactions between the conserved amino acids Q92 to G94 and the ligand, aided by a network of local hydrogen bonding contacts, are able to constrain into which direction the B‐loop will most likely protrude from the core and at the same time drive the tip of the loop away from the core. Whereas in the *presence* of the ligand, the (temporary) formation of these H‐bonds acts like a latch, directing the beginning and end toward one predominant side, the simulations nicely show that in the *absence* of the ligand, the stem of can flip over and samples a heterogeneous pattern of random orientations.) Residues 69–89, by contrast, show high sequence variation, hence giving further support to the kinetically driven model based on conformational preselection and avidity of multiple weak interactions. The intricacies of PstA have been prohibiting a more in‐depth biological assessment.^[^ ^14^ ^]^ Systems that are more tractable both from a structural‐biology as well as a cell‐biological perspective, like *E. coli* GlnK and its target, the ammonia channel AmtB (see Figure S2), may hence be useful to overcome the limitations of this study with respect to assays and mutation studies to verify the here assumed connectivity between the biophysical observations and the actual biological impact.

The allosteric modulation of spatial residence for overall flexible structural elements via concatenated transient interactions, amplified by a multitude of weak individual effects, adds to the emerging picture of a fine‐tuned interplay of disorder and order in conformational ensembles throughout the cell. Entirely (intrinsically) disordered proteins (IDPs) or long disordered regions (IDRs) terminal to well‐folded globular domains with a breadth of differentially stable and stabilizable structural properties, in particular secondary structural features, have seen soaring biophysical attention. The case of flexible loops within folded domains, with their ability to orchestrate interactions with interaction partners via allosteric modulation by events in the core via sequential short‐lived interactions hence appears as an important aspect that has not been specifically addressed in detail. It comes to no surprise, and has been well‐known, that short loops, as the T‐loop of this study, which practically folds directly *onto* the ligand here, can drastically change their motional properties (i.e., rigidify) upon ligand binding. By contrast, we have demonstrated here how the spatial properties of the dynamic ensembles formed by much longer loops and hence their interaction profiles and overall properties can be sensitively tweaked by alteration/creation of new sets of transient interactions and translation of the effect through coupled structural interdependencies and transient intramolecular interaction networks within a dynamic ensemble. This ensemble modulation represents another possibility—adding to the growing range of allosteric mechanisms being explored hitherto—to tailor functionality via spatially distant binding sites. Even though the effect of quaternary interactions, providing interaction surfaces that influence structural propensities of other proteins, is well known, the spatial redistribution of a conformational ensemble with retained dynamic properties via subtly redirecting, transient interactions will be a relevant and likely general addition to our understanding of how ensembles can be modulated.

## Conclusion

Here, we have shown how association of a secondary messenger to its receptor, for which discernable differences upon activation have been elusive due to the conformational plasticity of the relevant interaction sites, can change its biophysical properties through a network of transient interactions. Apart from quenching of µs timescale conformation exchange dynamics in the core, the association of the ligand is found to impact the spatial properties of the dynamic conformational ensemble formed by the 30 amino acids long loop thought to orchestrate the binding to downstream interaction partners. The subtle, indirect impact by which the properties of the dynamic ensemble are altered upon ligand binding to the protein core via a network of transient intra‐ and intermolecular interactions acts as an allosteric modulation of basic biophysical features and may be of interest for understanding the functional roles of disordered structural elements and their behavior as a function of upstream regulatory events generally.

## Supporting Information

The authors have cited additional references within the Supporting Information.^[^ ^48^, ^49^, ^50^, ^51^, ^52^, ^53^, ^54^, ^55^, ^56^, ^57^, ^58^, ^59^, ^60^, ^61^, ^62^, ^63^, ^64^, ^65^, ^66^, ^67^, ^68^, ^69^ ^]^

## Conflict of Interests

The authors declare no conflict of interest.

## Supporting information



Supporting Information

## Data Availability

The data that support the findings of this study are openly available in BMRB at www.bmrb.io, reference number 52882 and 52883.
