# Supplementary material for: Activation of a Secondary‐Messenger Receptor via Allosteric Modulation of a Dynamic Conformational Ensemble
Source: Angew Chem Int Ed Engl. 2025 Aug 6;64(38):e202509394. doi: 10.1002/anie.202509394 (PMC12435418; doi:10.1002/anie.202509394)
Supplement: Supplementary file 1 — Supporting Information [file ANIE-64-e202509394-s001.pdf]

Supporting Information for

Activation of a Secondary-Messenger Receptor via Allosteric Modulation of a Dynamic Conformational Ensemble

Benedikt Söldner#[1,2], Himanshu Singh#[1,2,3], Elias Akoury#,[2,4] Gregor Witte,[5] Rasmus Linser[1,2*]

[1] Faculty of Chemistry and Chemical Biology, Technical University Dortmund, Otto-Hahn Strasse 6, 44227 Dortmund, Germany

[2] Faculty of Chemistry and Pharmacy, Ludwig-Maximilians University, Butenandtstrasse 5-13, 81377 Munich, Germany

[3] Department of Biosciences and Bioengineering, Indian Institute of Technology Guwahati, Guwahati, 781039, Assam, India

[4] Department of Physical Sciences, Lebanese American University, Beirut 1102-2801, Lebanon

[5] Gene Center, Ludwig-Maximilians University, Feodor-Lynen Strasse 25, 81377 Munich, Germany

Experimental details: ***2***

Recombinant Protein Expression and Purification 2

NMR Backbone Assignment 2

Table S1: Experiments recorded for assignments. 3

Backbone ^{15}N Relaxation Experiments 3

Table S2: Exchange contribution and timescales 4

Molecular Dynamics Simulations 5

Protein Sequence Alignment 6

Small Angle X-ray Scattering 6

Isothermal Titration Calorimetry 6

Supplementary Figures ***7***

Fig. S1: Monomeric units of the crystal structures of PstA homologs 7

Fig. S2: Association of the homologue PII signal transduction protein GlnK of E. coli to its target 7

Fig. S3: Overall topology of PstA and B-loop hydrophobicity 8

Fig. S4: Energetics of binding of c-di-AMP to PstA 8

Fig. S5: Exemplary walk through the primary sequence 9

Fig. S6: Overlay of 2D HSQC spectra for apo PstA (blue) and c-di-AMP-PstA complex 9

Fig. S7: ^{15}N-edited NOESY strips for ligand:protein contacts 10

Fig. S8: Embedding of the c-di-AMP ligand as seen in crystallography 10

Fig. S9: RMSD of the classical MD (cMD) simulations 11

Fig. S10: Secondary structural propensities as seen in the MD simulation 11

Fig. S11: Projection of the cluster IDs resulting from clustering the B-loop 12

Fig. S12: Projection of the cluster IDs resulting from clustering the T-loop and core 12

Fig. S13: Peptide flip in residues 26 – 28 on the fast μs timescale 13

Fig. S14: Distribution of loop orientations 13

Fig. S15: Small-angle X-ray scattering curves 14

Fig. S16: Distribution of collective forward positioning 14

Fig. S17: Contact analyses for apo PstA and the PstA:c-di-AMP complex 15

Table S3: Most important hydrogen bonds formed in MD and their relative occurrence 15

Fig. S18: Conservation of the PstA primary sequence 16

Fig. S19: Simplified representation of the transient interactions 17

Fig. S20: H-bonds seen in the crystal structure of holo PstA of *L. monocytogenes* 17

References: ***18***

Experimental details:

Recombinant Protein Expression and Purification

A pET28a expression vector was used to clone *Staphylococcus aureus* PstA (SACOL0525) via NdeI and NotI restriction sites. Unlabeled, $^{1}H/^{15}N$-, $^{1}H/^{15}N/^{13}C$-, and $^{2}H/^{15}N/^{13}C$- uniformly labeled PstA constructs in conjunction with an N-terminal His_6-tag first without, later including a thrombin cleavage site (to avoid overlapping peaks in the central region of the H/N plane) were all expressed in *Escherichia coli* Rosetta BL21(DE3) cells and purified by Ni-NTA affinity and size exclusion chromatography (GE Healthcare) as the following: Cells were grown in protonated or deuterated M9 minimal medium containing ^{15}N-NH_4Cl and ^{13}C-glucose as sole sources of nitrogen and carbon, respectively. After reaching OD_{600} of 0.6, protein expression was induced by 1 mM IPTG and the cell culture was grown for 18 hours at 18ºC. Cells were harvested and re-suspended in lysis buffer A (50 mM Tris-HCl pH 7.5, 300 mM NaCl, 10 mM Imidazole, 10 % v/v glycerol) and flash frozen. Cells were then thawed, homogenized and cell lysates were centrifuged at 17000 g for 30 min at 4 ºC. The supernatant was incubated with His-Select Nickel affinity gel resin Ni-NTA (Qiagen) for 30 min at 4 ºC. After washing with buffer B (50 mM Tris-HCl pH 7.5, 300 mM NaCl, 40 mM Imidazole, 10 % v/v glycerol), buffer C (50 mM Tris-HCl pH 7.5, 300 mM NaCl, 300 mM Imidazole, 5 % v/v glycerol) was used to elute the protein from the resin. After an overnight dialysis with buffer D (50 mM Tris-HCl pH 7.5, 100 mM NaCl, 5 % v/v glycerol), anion exchange chromatography followed. PstA was loaded onto a HiTrap Q HP column (GE Healthcare) and eluted with a linear gradient to 100% of buffer E (50 mM Tris-HCl pH 7.5, 1 M NaCl, 5 % v/v glycerol). Samples without thrombin cleavage site were finally concentrated and loaded onto a HiLoad Superdex 75 column (GE Healthcare) equilibrated with buffer F (50 mM phosphate buffer, pH 6.8, 50 mM NaCl, 1 mM DTT) for further purification by size exclusion chromatography. For the construct with a thrombin cleavage site, the protein was incubated with thrombin at room temperature for 10 h for His-tag removal. Protein solutions with the cleaved His-tag were then concentrated and loaded onto a HiLoad Superdex 75 column (GE Healthcare) equilibrated with buffer F (50 mM phosphate buffer, pH 6.4, 50 mM NaCl, 1 mM DTT) for further purification by size exclusion chromatography. The resulting protein fractions were concentrated and analyzed by 15 % SDS-PAGE.

NMR Backbone Assignment

All NMR experiments were performed at 30 ºC on a Bruker Avance III 800 MHz spectrometer equipped with a cryogenic probe. A set of $^{1}H/^{15}N/^{13}C$ and $^{2}H/^{15}N/^{13}C$ PstA protein samples (0.5 mM in 50 mM phosphate buffer pH 6.8 or 6.4, respectively, 150 mM NaCl, 1 mM DTT and 10 % (v/v) D_2O) were used to measure three-dimensional (3D) HNCA, HNCACB, HNCO, HN(CA)CO, CBCA(CO)NH TROSY-based triple-resonance experiments for the NMR backbone assignments of free and c-di-AMP-bound forms (see details in Table S2). A 3D ^{15}N-NOESY-HSQC experiment was recorded with 2048 (F1) x 48(F2) x 256 (F3) complex points, 16 scans per increment with spectral widths of 16025 Hz, 4055 Hz, and 16025 Hz in the ^{1}H, ^{15}N, ^{1}H dimensions, respectively, and a total experiment time of 2.5 days.

NMR titrations contained 150 μM ^{15}N-labelled protein in the same buffer with increasing amount of c-di-AMP (Biolog) to achieve PstA:c-di-AMP ratios of 1:0.5, 1:1, 1:2 and 1:5. 2D ^{1}H-^{15}N TROSY-HSQC experiments were acquired using 600 complex points and 32 scans per increment with spectral widths of 14423 Hz and 2839 Hz in the ^{1}H and ^{15}N dimensions, respectively, and a total experiment time of 6 hours. All NMR experiments were processed with TopSpin (Bruker

BioSpin) and analyzed with CCPN Analysis.[1] Averaged chemical shift perturbations were calculated according to the equation $\Delta\delta_{tot} = \sqrt{\Delta\delta_H^2 + (0.14 \cdot \Delta\delta_N)^2}$.

For NMR relaxation studies, uniformly ^{15}N-labelled (u-^{15}N) and ^{13}C/^{15}N/^{2}H-triply-labelled (u-^{13}C/^{15}N) PstA samples were prepared in a mixed solvent of 90 % H_2O and 10 % ^2H_2O (50 mM sodium phosphate, 50 mM NaCl (pH = 6.4)). All NMR experiments were carried out at 30 ºC with protein concentrations between 0.5 and 0.6 mM on a Bruker Avance 800 MHz NMR spectrometer.

Table S1: Experiments recorded for assignments. The index $^{\mathbf{His}}$ denotes samples of a first iteration with a His$_6$-tag at pH 6.8.

Sample	Spectrum	Dimension			Points			Scans	Spectral width (ppm)		
		F1	F2	F3	F1	F2	F3		F1	F2	F3
Apo	^{15}N-HSQC	H	N		4096	256		16	12500	2839	
Apo^{His}	^{15}N-TROSY	H	N		2048	512		16	14423	2839	
Holo^{His}	^{15}N-TROSY	H	N		2048	512		40	14423	2839	
Apo	^{15}N-TROSY	H	N		2048	128		8	14706	2839	
Holo	^{15}N-TROSY	H	N		2048	256		16	14706	2839	
Holo^{His}	CBCA(CO)NH	H	N	C	2048	64	220	32	11161	2839	16077
Apo	CBCA(CO)NH	H	N	C	2048	48	128	32	11364	2839	16100
Apo^{His}	HN(CA)CO	H	N	C	2048	120	128	16	11161	2839	2818
Holo^{His}	HN(CA)CO	H	N	C	2048	128	128	16	11161	2839	2818
Holo	HN(CA)CO	H	N	C	2048	48	128	8	11364	2839	2818
Apo^{His}	HNCA	H	N	C	2048	48	230	16	11161	2839	6039
Holo^{His}	HNCA	H	C	N	2048	120	40	8	14423	6039	2839
Apo	HNCA	H	N	C	2048	64	128	16	11364	2839	6037
Holo	HNCA	H	N	C	2048	48	110	8	11364	2839	6037
Apo^{His}	HNCACB	H	N	C	2048	120	144	16	14423	2839	16077
Holo^{His}	HNCACB	H	N	C	2048	120	144	16	14423	2839	16077
Apo	HNCACB	H	N	C	2048	124	128	16	14706	2839	16100
Holo	HNCACB	H	N	C	2048	110	128	16	14706	2839	16100
Apo^{His}	HNCO	H	N	C	2048	40	108	16	14423	2839	2818
Holo^{His}	HNCO	H	N	C	2048	114	56	8	14423	2839	2818
Holo	HNCO	H	N	C	2048	40	128	8	12500	2839	3220

Backbone ^{15}N Relaxation Experiments

^{15}N Longitudinal (R_1) and transverse (R_2) relaxation rates and steady-state ^{1}H-^{15}N heteronuclear Nuclear Overhauser Effect (hetNOE) experiments on ^{15}N spins of the backbone were performed on apo and c-di-AMP-bound PstA at a B_0 field of 18.8 T and 30 ºC. ^{15}N backbone relaxation rates R_1 and R_2 were measured using relaxation delays of 1, 10, 50, 100, 200, 300, 600, 800, and 1000 ms for R_1 and 17.0, 33.9, 50.9, 67.8, 84.8, 101.8, 135.7, 152.6, 169.6, 203.5, and 254.4 ms for R_2. Peak intensities were fitted to a single exponential decaying function. ^{1}H-^{15}N hetNOE intensities were

measured in an interleaved manner using ^{1}H saturation time of 3 s and a recycle delay of 5 s, which was adequate to completely saturate amide proton resonances. Steady-state [^{15}N, ^{1}H] heteronuclear-NOE measurements were carried out with and without proton saturation during the relaxation delay. In these NOE-experiments, 5 s of relaxation delay and 3 s of proton saturation (or 8 s of relaxation delay only) were used. The heteronuclear-NOE values were determined as the ratio of the peak intensities measured from the spectra acquired with and without proton saturation. All relaxation measurements were performed at 303 K. NMR spectra were processed using TopSpin (Bruker BioSpin) and analyzed using CARA[2] and CCPN[1]. The added contribution to the dephasing seen for the R_2 rates of the apo form compared to the holo receptor cannot stem from processes slower than the µs timescale, as those would be reflected in the BMRD experiments and as the R_2 rates were recorded using a CPMG pulse train of 2 kHz.

Constant-time ^{15}N-CPMG (CT-CPMG) relaxation dispersion experiments[3] were measured at 303 K. Experiments were performed with a constant-time delay of 40 ms, and 9 variable CPMG frequencies (ν_{CPMG}) ranging from 50 to 2000 Hz were collected. In addition, a reference spectrum without constant time delay ($T_{\mathrm{CPMG}} = 0$) was recorded. Each 2D dataset corresponding to one CPMG frequency, 128 and 2048 complex points in the indirect and direct dimensions, respectively, was collected with 64 scans. 2D datasets for all frequencies were measured in a scan-interleaved fashion. The recycle delay of 1.5 s was used, giving rise to a net acquisition time of approximately 5.2 h per data set. The ^{15}N carrier during the CPMG period was set to 118 ppm and 117 ppm for PstA in the apo and ligand-bound form, respectively. The ^{15}N 90° pulse width of CPMG pulses was set to 32.0 µs. NMR spectra were processed using TopSpin (Bruker BioSpin). Peak intensities were quantified and visualized with CCPN[1]. The effective amide ^{15}N transverse relaxation rate at each CPMG frequency was calculated according to the relation of effective transverse relaxation rate

$$R_{2,\mathrm{eff}} = -\frac{1}{T_{\mathrm{CPMG}}} \cdot \ln\left(\frac{I}{I_0}\right)$$

where I is the peak intensity, I_0 is the corresponding intensity in a reference spectrum recorded without the CT-CPMG relaxation period and T_{CPMG} is a constant time delay. Evaluation of CPMG data was performed using ChemEx[4]. Relaxation dispersion curves were fitted analytically according to the Block-McConnell equations on the basis of a two-state exchange model in a residue-specific and, in addition, for several residues showing relaxation dispersion also in a global manner. The noise level of each spectrum was used as error for the intensities and propagated in the fitting procedure. The exchange rate R_{ex} was determined as difference between the maximum calculated effective transverse relaxation rate $R_{2,\mathrm{eff}}$ (maximum of the fit curve) and the fitted intrinsic transverse relaxation rate R_2^0.

Table S2: Exchange contribution and timescales of the individual relaxation dispersion fits (for residues with significant exchange only).

Residue	τ_{ex}	R_{ex}
**27**	(5.7 ms ± 38.1 ms)	18.5 Hz ± 2.3 Hz
**28**	166.2 µs ± 83.4 µs	15.2 Hz ± 3.5 Hz
**29**	(222.0 µs ± 819.7 µs)	16.5 Hz ± 8.7 Hz
**32**	38.6 ms ± 8.9 ms	37.5 Hz ± 3.8 Hz
**34**	(10.7 ms ± 60.3 ms)	14.5 Hz ± 2.8 Hz
**46**	166.4 µs ± 67.5 µs	20.0 Hz ± 4.5 Hz

A global fit (of $k_{\text{ex,AB}}$ and population P_{B}) of residues 28, 29 and 46 yields $k_{\text{ex}} = 190.8\ \mu\text{s} \pm 94.1\ \mu\text{s}$, i.e., a 100 µs timescale conformational exchange. However, while the presence/absence of exchange is unambiguous, all of these timescales of the individual dispersion profiles are difficult to fit in an accurate fashion due to the low signal to noise obtained for the trimeric complexes.

Molecular Dynamics Simulations

All MD simulations were performed using the CHARMM36m force field[5], which is well established for both ordered and disordered proteins, together with the TIP3P water model.

For the Gaussian-accelerated molecular dynamics simulations performed with NAMD 2.14[6], starting structures obtained from clustering of preliminary, not further described, classical MD simulations of PstA apo and ligand bound form were used. These starting structures differed in each chain and the B-loops were in extended conformation close to the protein surface and did not contain any well-defined secondary structure elements. Both systems were prepared using CHARMM-GUI[7]. Both systems were placed into an octahedral box with unit cell lengths of 10.5 nm, solvated with TIP3P water[8] at pH 7 and neutralized with NaCl at a physiological concentration of 50 mM. After energy minimization, the respective system was equilibrated for 500 ps in the NVT ensemble and for 2.5 ns in 5 steps in the NpT ensemble, during which the position restraints were gradually released. Temperature was adjusted to 303.15 K using the Langevin thermostat and the pressure was kept constant at 1 bar using the Nosé-Hoover Langevin piston barostat[9]. All bond lengths in protein and ligand were kept constant with SHAKE, allowing to integrate the equations of motion with 2 fs time steps. Lennard-Jones 12-6 and electrostatic interactions were smoothly shifted to zero at a 1.2 nm cut-off. Long-range electrostatic interactions were treated with the particle mesh Ewald algorithm[10]. The dual-boost biasing potential (on both, dihedral and total potential energy) was equilibrated in two steps[11]: At first, 10 ns of conventional MD were performed, in which statistics about the potential energy of the system were collected. In the second 50 ns long step, collection of potential statistics was continued, but an additional, concurrently updated biasing potential was applied. In the end of the equilibration, the potential statistics were fixed for calculating the boost potential applied at the production run. The upper limit $\sigma_{0\text{D/P}}$ for the standard deviation of the potential energy was set to 18 kcal/mol, which corresponds to acceleration factors k_0 of $\sim$0.9 and $\sim$0.1 for the dihedral and total boost potential. The final Gaussian accelerated production runs were conducted for 1 µs. Although this high biasing potential does not allow for accurate reweighting of the potential energy surface anymore, it still ensures that the large conformational space of the B-loop is extensively sampled and provides more reliable and representative starting structures for replica MDs than simple loop modelers, which are only suited for short and rather rigid loops.[12]

The classical MD simulations of PstA in apo and c-di-AMP-bound form were carried out with Gromacs 2024.2[13]. As starting structures, snapshots were taken every 200 ns from the preceding GaMD simulation. After energy minimization, the respective system was equilibrated for 500 ps in the NVT ensemble and for 2.5 ns in 5 steps in the NpT ensemble, during which the position restraints were gradually released. Temperature was kept constant at 303.15 K using the V-rescale thermostat developed by Bussi and coworkers[14] and pressure was kept constant at 1 bar using the Parrinello-Rahman barostat[15]. All bond lengths in protein and ligand were constrained with LINCS, and water molecules were kept rigid with SETTLE, allowing to integrate the equations of motion with 2 fs time steps. Lennard-Jones 12-6 interactions were smoothly shifted to zero at a 1.2 nm cut-off. Long-range electrostatic interactions were

treated with the particle mesh Ewald algorithm[10] using a distance of 1.2 nm for switching between short- and long-range electrostatic interactions. The final production runs were performed for 1 µs each.

Protein Sequence Alignment

Protein sequence alignment was generated by the constraint-based alignment tool COBALT[16] for 50 PstA homologues, including *Staphylococcus aureus* PstA (PDB codes 4WK1 and 4D3H), *Listeria monocytogenes* PstA (PDB code 4RWW) and *Bacillus subtilis* DarA (PDB code 4RLE) (compare Fig. S1).

Small Angle X-ray Scattering

Small angle X-ray scattering data were collected at EMBL DESY (Hamburg, Germany). PstA was additionally purified by size exclusion chromatography in tris buffer to ensure a sample free of aggerates. The c-di-AMP-bound PstA complex was formed after addition of filtered c-di-AMP in identical buffer conditions to the apo protein. To ensure absence of free c-di-AMP that might interfere with the buffer reference, we used a minimal under-saturation (99 %) for complex preparation. Samples were centrifuged prior to measurement and were measured in different concentrations, alternating with two buffer measurements for each sample in batch mode. After buffer correction, scattering data of the different concentrations were analyzed and merged using ATSAS[17] as previously described.[18] The radius of gyration R_g was determined by Guinier plot analysis ($\ln I(s)$ vs. s^2) including points with $s \cdot R_\mathrm{g} < 1.3$ in the linear regression (Guinier approximation for globular proteins) and pair-distribution analysis was performed using the GNOM option in ATSAS.[19] The Guinier plots for apo and c-di-AMP complex indicate that the samples are free of aggregates with $R_\mathrm{g}^{\text{apo-PstA}} = 2.67 \pm 0.01$ nm and $R_\mathrm{g}^{\text{PstA-c-di-AMP}} = 2.60 \pm 0.01$ nm. The error distribution shows that the sample is free of aggregates. Note that for the SAXS measurements of both, apo and holo receptor, samples with a His-tag were employed. While there is consistency within the SAXS samples and their relative differences are fully meaningful, a more quantitative comparison to the MD data or even a reweighting/restraining of the MD simulation would not have been correct. In this context, we note that even though this will incur quantitative differences for R_g values between the approaches – it does not have a bearing on the qualitative interpretations, as the His-tag is on the backside of the protein is far away from the B-loop. An influence of the His-tag on the dynamics of the B-loop (or vice versa a change of the His-tag dynamics) can be neglected. SAXS data were deposited into the Small Angle Scattering Biological Data Bank (SASBDB) under accession codes SASDXD5 and SASDXE5 for apo and holo PstA, respectively.

Isothermal Titration Calorimetry

Isothermal Titration Calorimetry (ITC) data were collected at 20 °C using a Malvern PEAQ-ITC system with 15 µM PstA in tris buffer in the cell. c-di-AMP was dissolved in the same buffer to 177 µM and titrated into the cell by 19 injections of 2 µl, spaced 150 s apart. Data evaluation was done using the Malvern PEAQ-ITC software. Experiments were repeated at least once to confirm the robustness of the assay.

Supplementary Figures

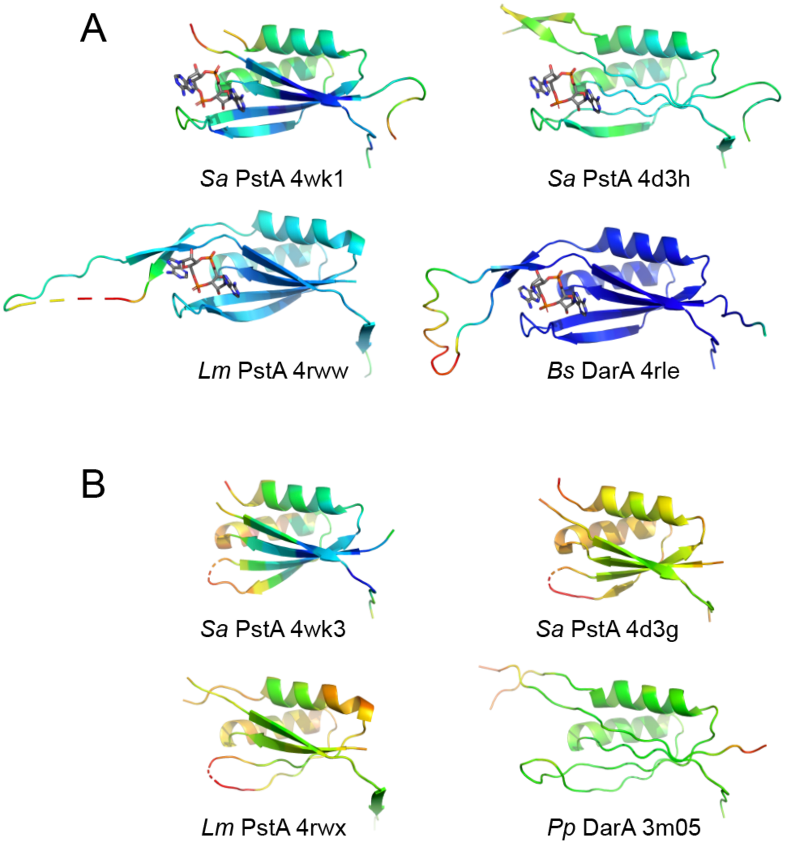

Fig. S1: Monomeric units of the crystal structures of PstA homologs. **A)** c-di-AMP-bound species, in particular PstA from *Staphylococcus aureus* (*Sa*) and *Listeria monocytogenes* (*Lm*), as well as DarA from *Bacillus subtilis* (*Bs*). **B)** Apo species, in particular again PstA from *S. aureus* and *L. monocytogenes*, as well as DarA from *P. pentosaceus* (*Pp*). Brackets denote the respective PDB entries. Colors are according to relative B-factor coloring in Pymol (blue: highly defined density, low density). The B-loop protrudes from the upper left.

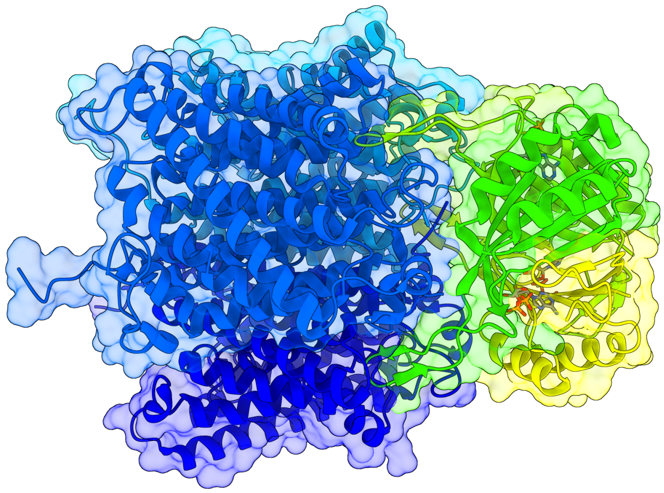

Fig. S2: Association of the homologue P_{II} signal transduction protein GlnK of *E. coli* to its target, the ammonia channel AmtB (PDB 2NUU) mediated by the three long T-loops.[20]

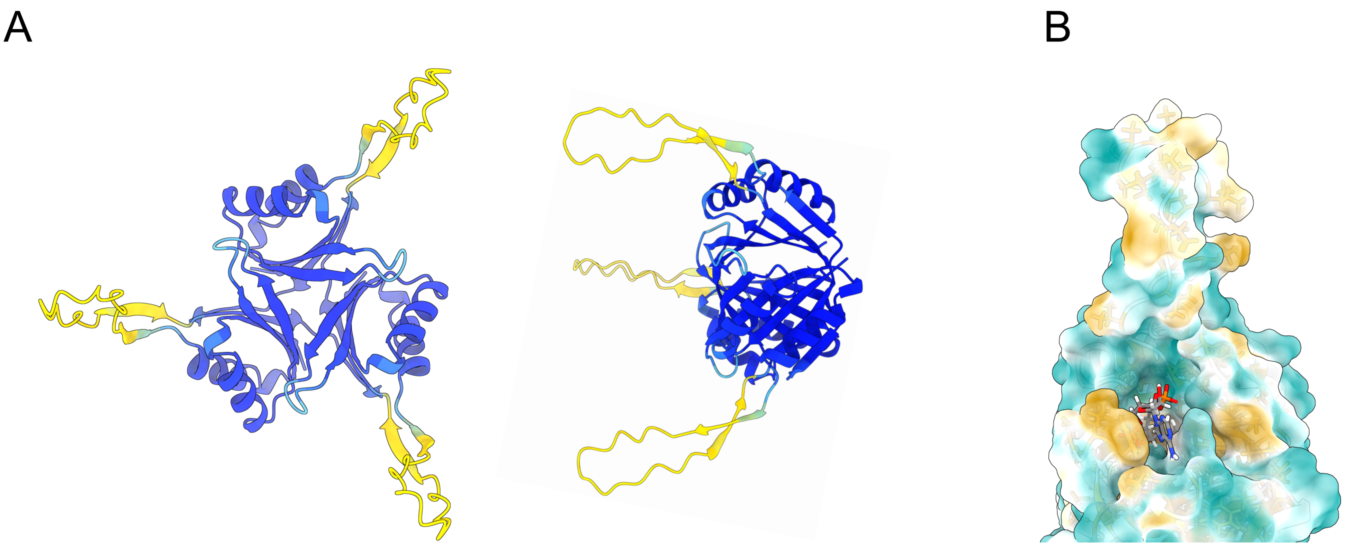

Fig. S3: Overall topology of PstA and B-loop hydrophobicity. **A)** AlphaFold structure of the *S. aureus* PstA monomer, showing the confidence of structural predictions as a color gradient (blue: confident, yellow: not confident). **B)** Hydrophobic surface of PstA in ligand-bound form. The B-loop is modelled here as extended structure ranging into the solvent. A high number of hydrophobic residues in the B-loop suggest potential interactions to the underlying surface (the ligand binding site), which is the only other region on the protein surface with high hydrophobicity.

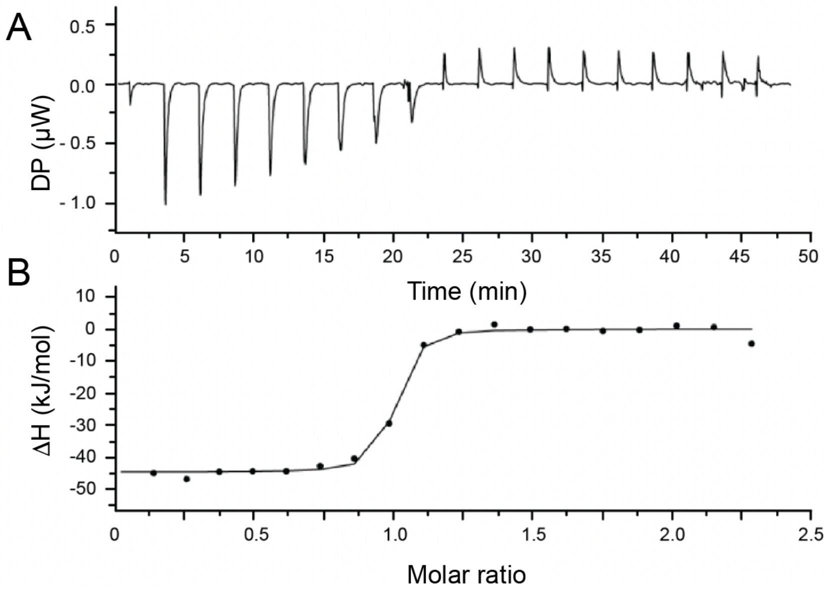

Fig. S4: Energetics of binding of c-di-AMP to PstA. **A)** Isothermal Titration Calorimetry (ITC) analysis of PstA (15 µM) with c-di-AMP (177 µM) monitored at 20 °C. **B)** Binding isotherm of the calorimetric titration of c-di-AMP into PstA, showing the integrated heats (dots) and the respective fit (solid line). The fitting marks a K_{D} of 19 ± 8 nM, $n = 0.95$, $\Delta H = -44.7$ kJ mol^{-1}).

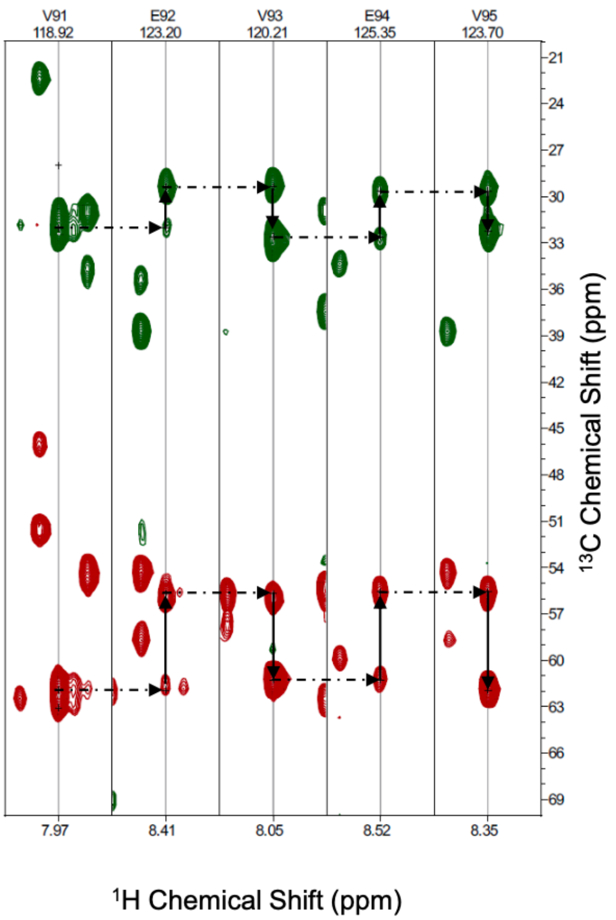

Fig. S5: Exemplary walk through the primary sequence, shown here for an HNCACB experiment for residues V91 to V95.

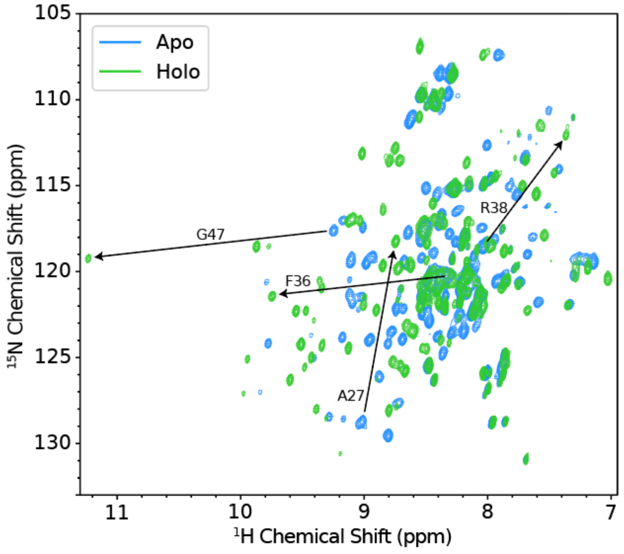

Fig. S6: Overlay of 2D [^{1}H, ^{15}N]-HSQC spectra for apo PstA (blue) and c-di-AMP-PstA complex (green) under similar experimental conditions, together with residue assignments containing CSPs > 1.0 ppm.

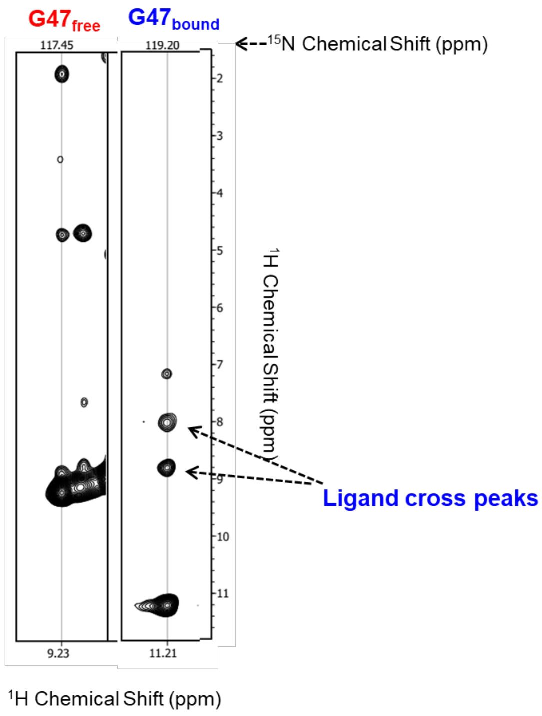

Fig. S7: ^{15}N-edited NOESY strips for ligand:protein contacts in close proximity to G47.

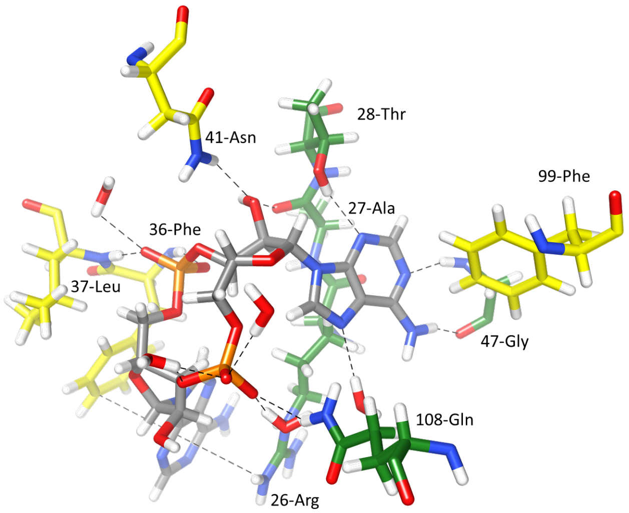

Fig. S8: Embedding of the c-di-AMP ligand as seen in crystallography. Hydrogen bonds and π interactions between c-di-AMP and PstA (PDB code 4wk1) in the binding cavity are shown as dashed and solid lines, respectively. Residues from monomer A are shown with green carbon atoms and residues from monomer B are shown with yellow carbon atoms. T28, L37, F38, N41, G47 and Q108 form hydrogen bonds with different parts of c-di-AMP, R26 forms a cation-π interaction with adenine, F36 a face-to-face π-stacking with adenine and F99 might form a weak edge-to-face π-stacking with adenine (in the crystal structure of SaPstA according to I. Campeotto et al., PDB code 4d3h[21]).

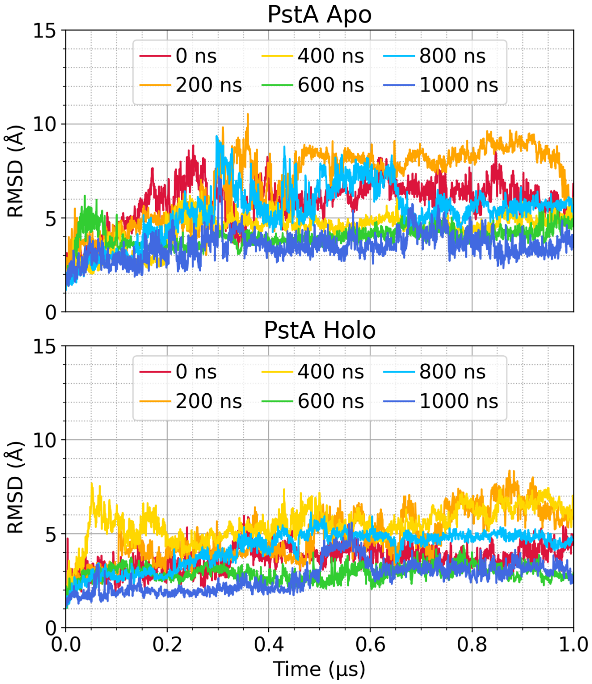

Fig. S9: RMSD of the classical MD (cMD) simulations to the respective starting structure as a function of time. As these starting structures were obtained from initial GaMD simulations, the individual cMD runs (different colors) are labeled according to the timepoint at which they were initially found in the GaMD.

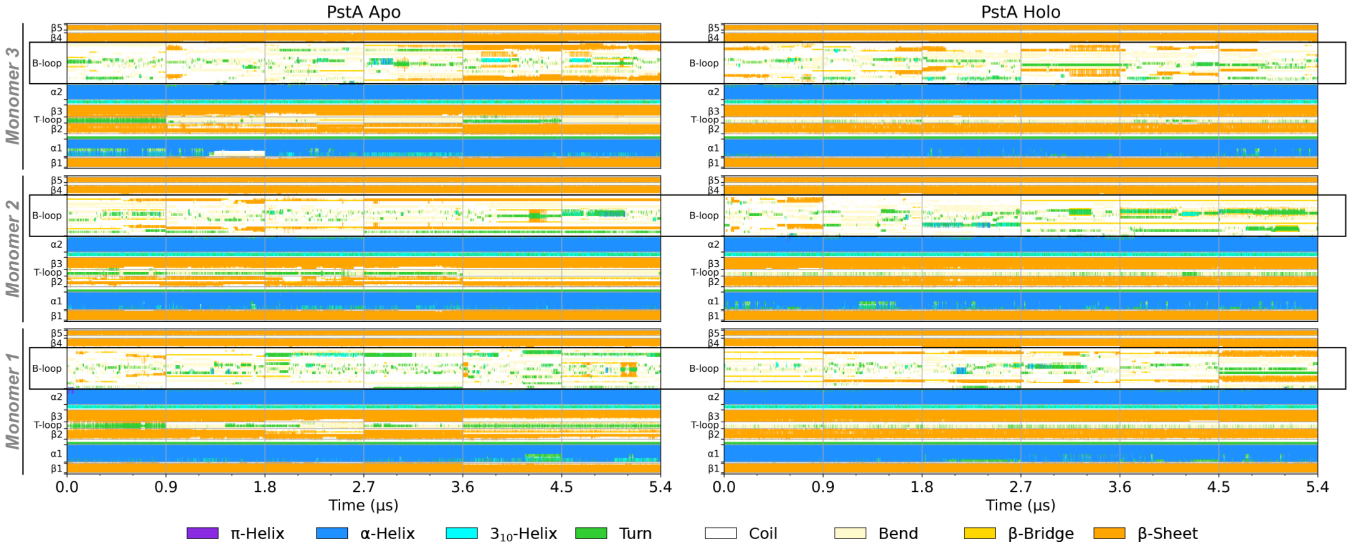

Fig. S10: Secondary structural propensities as seen in the MD simulation for apo (left) and holo form (right). The three monomers are plotted on top of each other, the individual trajectories (omitting the first 100 ns) are shown one after the other. The secondary-structure properties are denoted by colors as shown at the bottom.

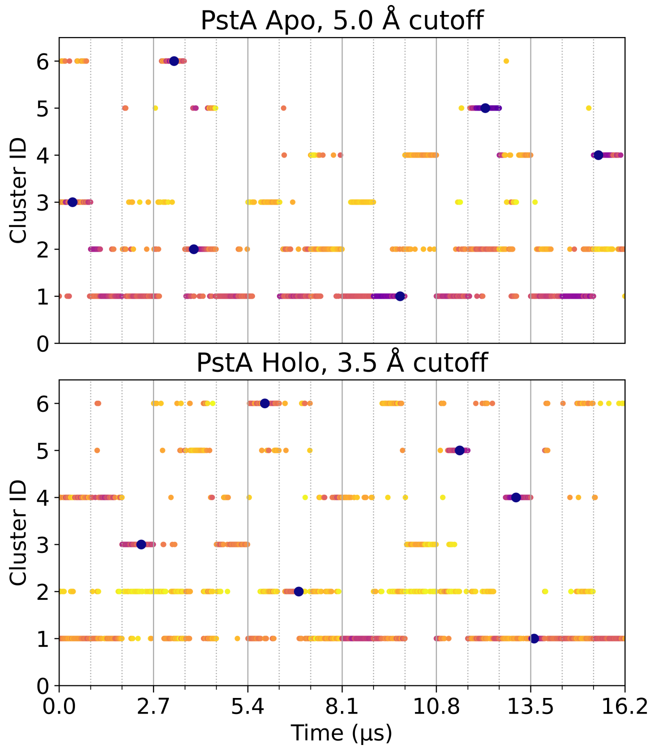

Fig. S11: Projection of the cluster IDs resulting from clustering of the concatenated trajectories of the extracted monomers (6 · 3 · 900 ns per trajectory analyzed) with respect to the simulation time using RMSD cutoffs of 5.0 Å and 3.5 Å for the apo and holo receptor. The dark blue dots indicate the cluster center and the coloring denotes the RMSD with respect to the respective cluster center. The high number of transitions between the different clusters substantiates a sufficient sampling of the performed conventional MD simulations and a low to negligible bias of the starting structures.

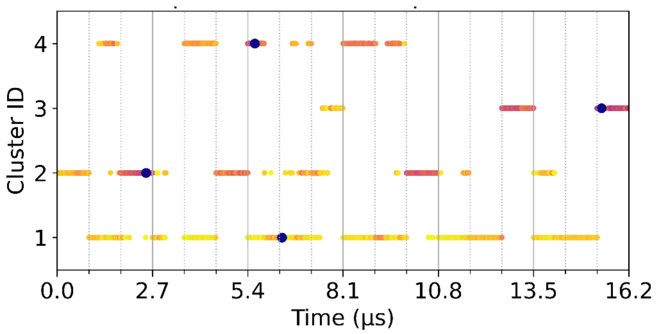

Fig. S12: Projection of the cluster IDs resulting from clustering the T-loop and core (disregarding B-loop conformations, with a cutoff of 1.2 Å), as in Main Text Fig. 4D. Represented in similar way as Fig. S11.

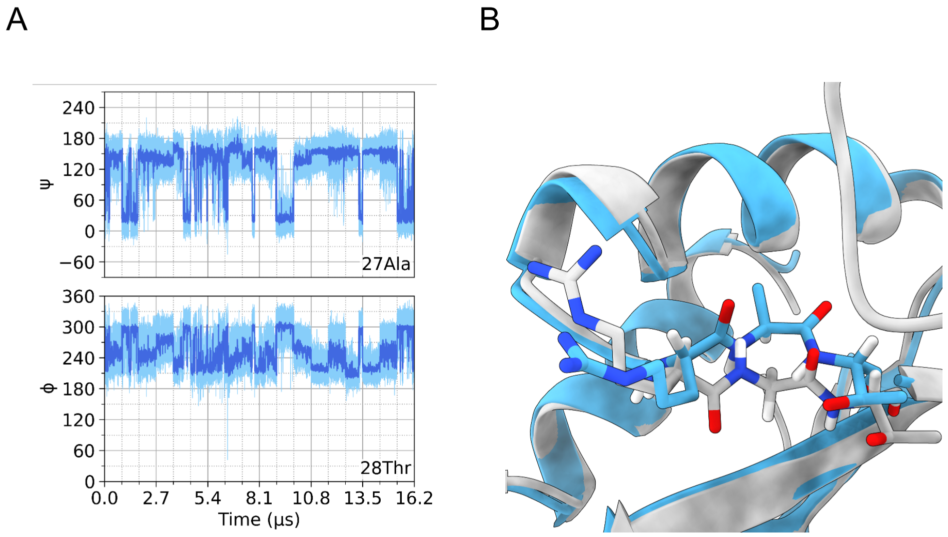

Fig. S13: Peptide flip in residues 26 – 28 on the fast μs timescale. This local motion is in addition to the chemical exchange witnessed for the entire region (27 – 34) in the relaxation dispersion experiments, which can only capture exchange phenomenons > 100 μs.

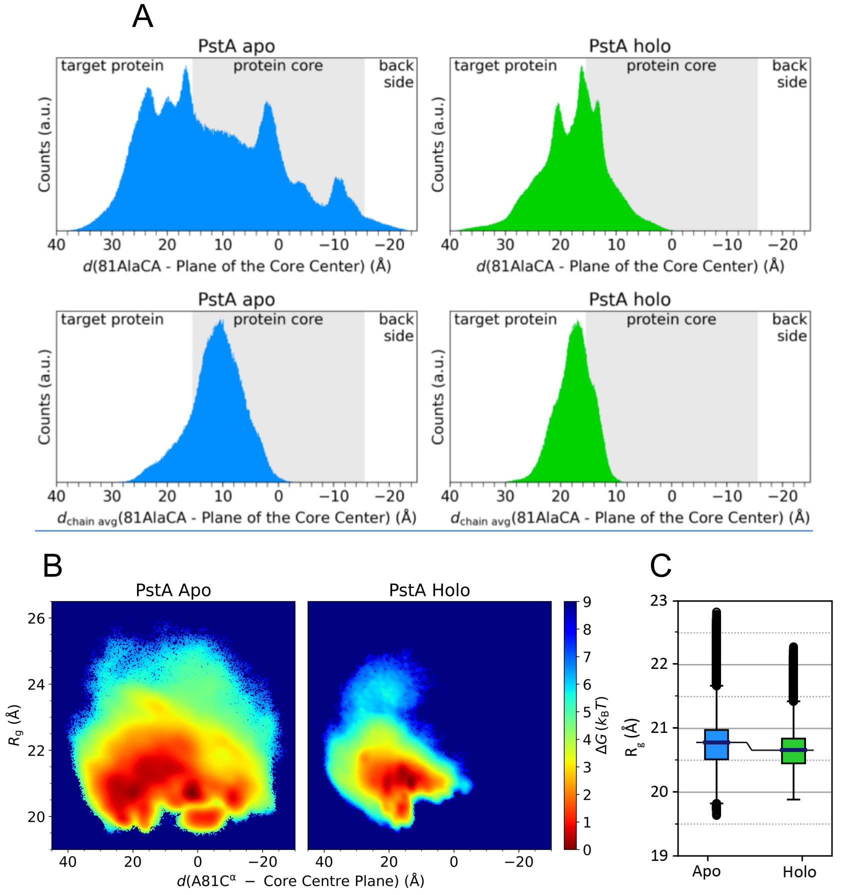

Fig. S14: Distribution of loop orientations. **A)** Distribution of individual (top) and chain-averaged loop orientations (below). **B)** Free energy surface of the individual orientations together with the corresponding radius of gyration (determined with respect to the center of mass of the trimer). **C)** Average radius of gyration (R_g) as obtained from the simulation for apo and holo PstA.

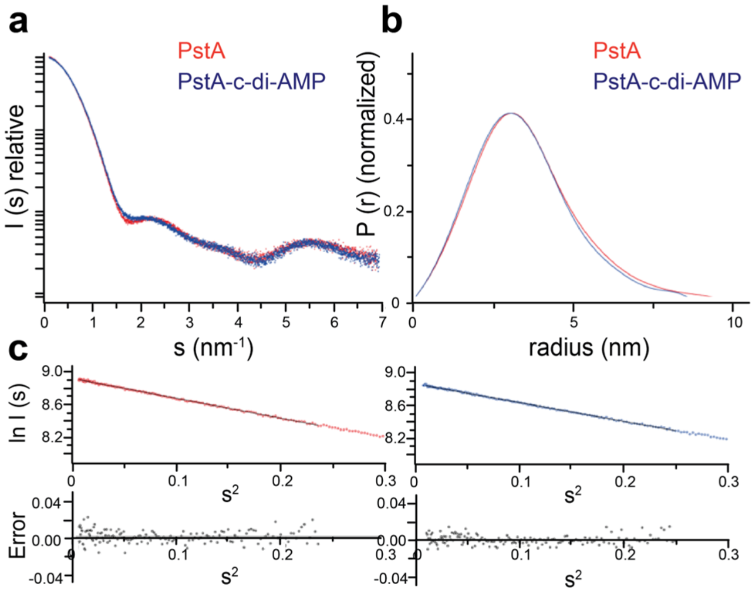

Fig. S15: Small-angle X-ray scattering curves of c-di-AMP-bound (blue) and free PstA (red). Despite similar overall shape, the complex shows differences in the local minimum at $s = 1.7\ \text{nm}^{-1}$. Pair distribution functions obtained using GNOM of PstA (red) and the complex (blue) indicate that the latter has a reduced maximum particle diameter ($D_{\text{max}}^{\text{PstA apo}} = 9.24$ nm; $D_{\text{max}}^{\text{PstA holo}} = 8.59$ nm). The error is estimated from the linear regression vs. the measured data. Accordingly, the Guinier plots are fitted to an experimental radius of gyration R_g of 2.67 ± 0.01 nm and 2.60 ± 0.01 nm for apo and holo form, which qualitatively matches with the simulation (Fig. S14C) and again confirms the modulation of overall loop redirection towards a more restricted space.

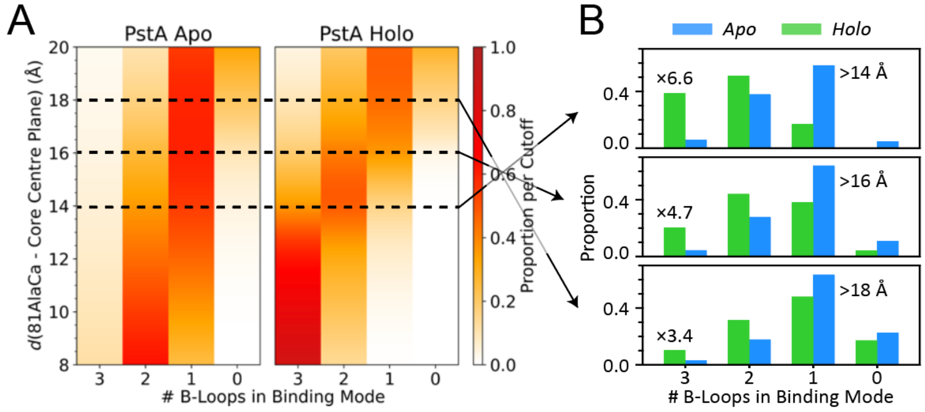

Fig. S16: Distribution of collective forward positioning (3, 2, 1, or none of the loops are beyond a given threshold) as a function of the threshold (minimum distance between A81 and the center of mass) chosen. In A), a continuous variation of the distance threshold is chosen, whereas in B), three discrete thresholds (14, 16, and 18 Å, same as in Main Text Fig. 5C) are specifically read out.

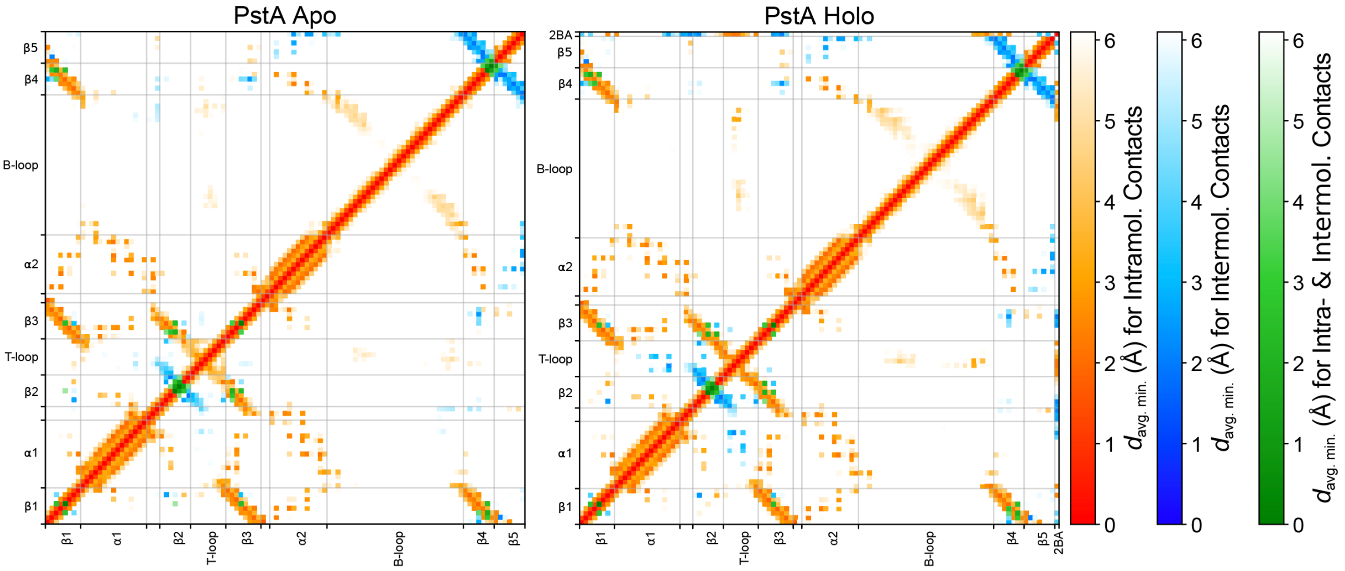

Fig. S17: Contact analyses for apo PstA and the PstA:c-di-AMP complex. Intramonomer contacts are shown in orange/red colors, intermonomer contacts are shown in blue colors, green denotes the presence of both, intra- and intermonomer contacts. PstA:c-di-AMP contacts (shown as the rightmost column/top row in the plot for the holo form) distinguish contacts to two different PstA monomers.

Table S3: Most important hydrogen bonds formed in MD and their relative occurrence.

H-bond			Percent of time H-bond exists		Type
			Apo	Holo	
2LysHZ*	–	104AspOD*	68 %	72 %	Core – Core intermol.
67ArgHH*	–	12AspOD*	57 %	67 %	B-loop – Core
95GlyO	–	9GlnH	18 %	64 %	B-loop – Core
95GlyH	–	109PheOT*	23 %	52 %	B-loop – Core intermol.
65GlyH	–	12AspOD*	64 %	50 %	B-loop – Core
38ArgHH*	–	18AspOD*	12 %	50 %	T-loop – Core intermol.
94GlyO/95GlyO	–	9GlnHE*	27 %	34 %	B-loop – Core
35GlyH	–	27GlyO	2 %	28 %	T-loop – Core intermol.
79GlyH	–	36PheO	0 %	14 %	B-loop – T-loop
34GlyO	–	29LysH	19 %	0 %	T-loop – Core intermol.
34GlyO	–	28ThrOD1	19 %	0 %	T-loop – Core intermol.
40GlyO	–	10AspH	36 %	1 %	T-loop – Core
2BA.N1	–	47GlyH	-	91 %	Ligand – Core
2BA.P*P1	–	36PheH	-	89 %	Ligand – T-Loop
2BA.O2P1	–	37LeuH	-	75 %	Ligand – T-Loop
2BA.H6*	–	47GlyO	-	68 %	Ligand – Core
2BA.H61*	–	25PheO	-	60 %	Ligand – Core
2BA.O2P	–	94GlyH	-	49 %	Ligand – B-Loop
2BA.N3	–	28ThrHG1	-	40 %	Ligand – Core
2BA.HO2'	–	28ThrOG1	-	38 %	Ligand – Core
2BA.H61*	–	21ValO	-	38 %	Ligand – Core
2BA.HO21	–	92GluO	-	37 %	Ligand – B-Loop
2BA.O1P1	–	35GlyH	-	29 %	Ligand – T-Loop
2BA.O1P	–	26ArgHH11	-	27 %	Ligand – Core
2BA.O2P	–	95GlyH	-	26 %	Ligand – B-Loop
2BA.O2'	–	28ThrHG1	-	25 %	Ligand – Core

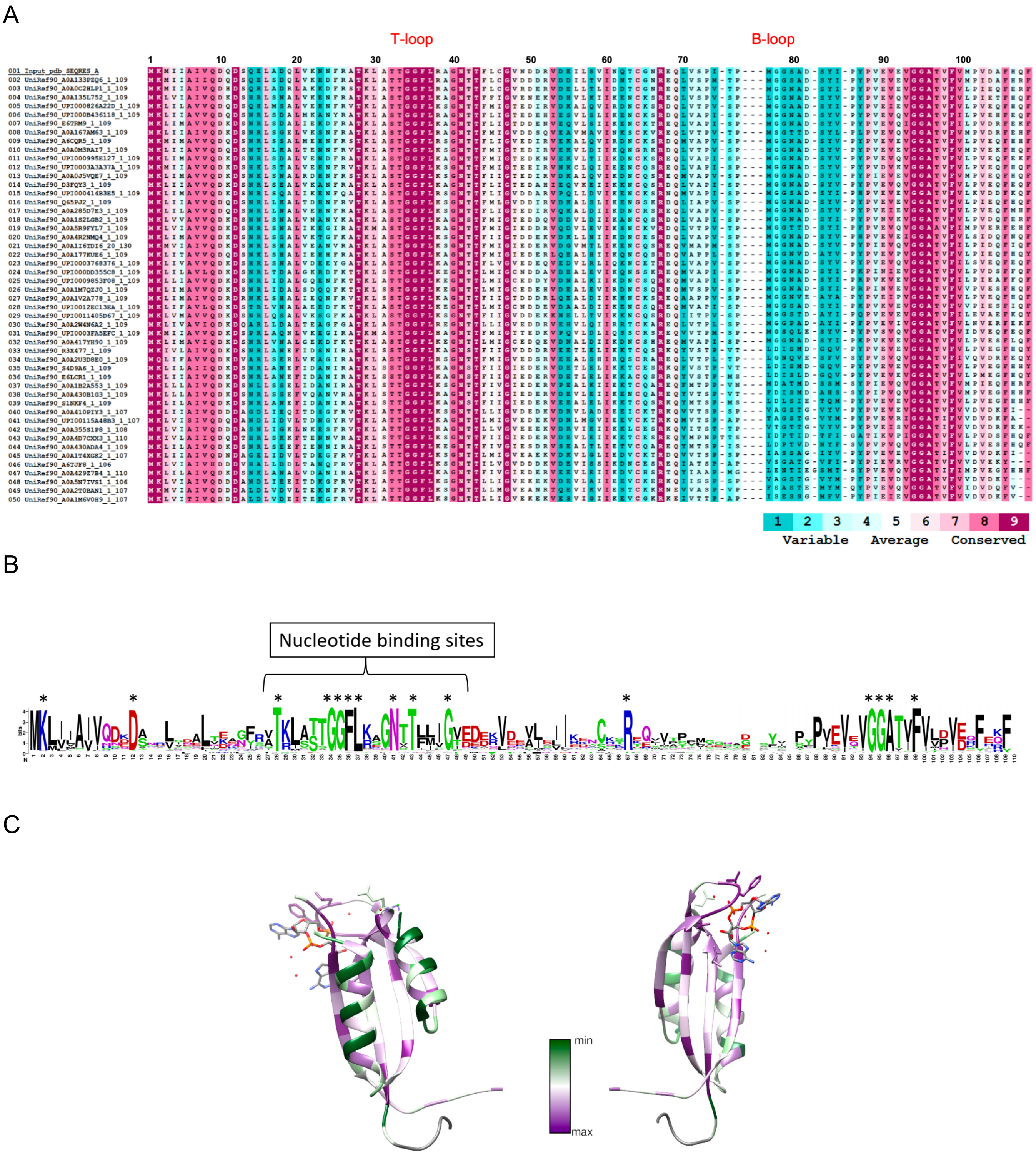

Fig. S18: Conservation of the PstA primary sequence. **A)** Alignment of all sequences compared. **B)** Conservation represented by the size of the respective letters in the primary sequence. **C)** Conservation color-coded on the monomeric, holo PstA structure.

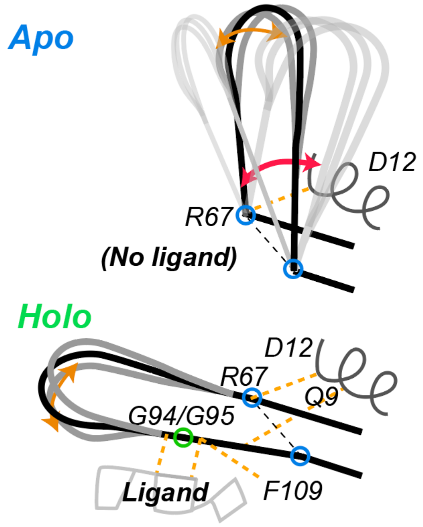

Fig. S19: Simplified representation of the transient interactions found to effectively redirect the B-loop conformational ensemble in the presence of the ligand. Whereas the salt bridge between R67 and D12 acts like a hinge (blue circles) both, in the apo and holo form, the newly arising network of transient interactions with and around the ligand acts like a latch (green) that effectively reduces angular motion of the loop at its root.

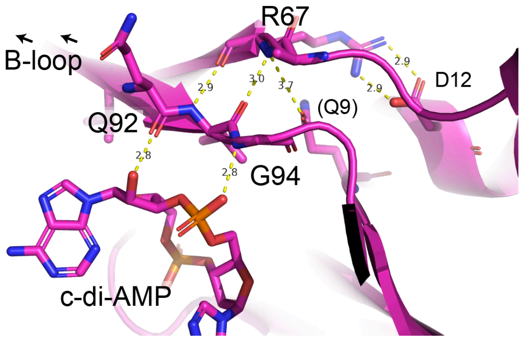

Fig. S20: H-bonds seen in the crystal structure of holo PstA of *L. monocytogenes* (PDB 4RWW). The stabilization of the loop's beginning and end upon crystallization, in particular between c-di-AMP and conserved residues Q92 and G94 at the end of the B-loop, as well as the salt bridge from the sidechain of R67 to D12 at the end of helix 1, seen here as a static architecture, coincides at least with part of the transient H-bonds seen in the dynamic conformational ensemble of the MD simulations.

References:

1. Vranken, W. F.; Boucher, W.; Stevens, T. J.; Fogh, R. H.; Pajon, A.; Llinas, P.; Ulrich, E. L.; Markley, J. L.; Ionides, J.; Laue, E. D., The CCPN data model for NMR spectroscopy: development of a software pipeline. *Proteins* **2005,** *59*, 687–696.
2. Keller, R. Optimizing the process of nuclear magnetic resonance spectrum analysis and computer aided resonance assignment. PhD thesis, ETH Zurich, 2004.
3. Mulder, F. A. A.; Skrynnikov, N. R.; Hon, B.; Dahlquist, F. W.; Kay, L. E., Measurement of Slow (μs−ms) Time Scale Dynamics in Protein Side Chains by 15N Relaxation Dispersion NMR Spectroscopy: Application to Asn and Gln Residues in a Cavity Mutant of T4 Lysozyme. *J. Am. Chem. Soc.* **2001,** *123* (5), 967-975.
4. Bouvignies, G. https://github.com/gbouvignies/ChemEx.
5. Huang, J.; Rauscher, S.; Nawrocki, G.; Ran, T.; Feig, M.; de Groot, B. L.; Grubmüller, H.; MacKerell, A. D., CHARMM36m: an improved force field for folded and intrinsically disordered proteins. *Nat. Methods* **2017,** *14* (1), 71-73.
6. Phillips, J. C.; Hardy, D. J.; Maia, J. D. C.; Stone, J. E.; Ribeiro, J. V.; Bernardi, R. C.; Buch, R.; Fiorin, G.; Hénin, J.; Jiang, W.; McGreevy, R.; Melo, M. C. R.; Radak, B. K.; Skeel, R. D.; Singharoy, A.; Wang, Y.; Roux, B.; Aksimentiev, A.; Luthey-Schulten, Z.; Kalé, L. V.; Schulten, K.; Chipot, C.; Tajkhorshid, E., Scalable molecular dynamics on CPU and GPU architectures with NAMD. *J. Chem. Phys.* **2020,** *153* (4).
7. (a) Jo, S.; Kim, T.; Iyer, V. G.; Im, W., CHARMM-GUI: A web-based graphical user interface for CHARMM. *J. Comput. Chem.* **2008,** *29* (11), 1859-1865; (b) Lee, J.; Cheng, X.; Swails, J. M.; Yeom, M. S.; Eastman, P. K.; Lemkul, J. A.; Wei, S.; Buckner, J.; Jeong, J. C.; Qi, Y.; Jo, S.; Pande, V. S.; Case, D. A.; Brooks, C. L., III; MacKerell, A. D., Jr.; Klauda, J. B.; Im, W., CHARMM-GUI Input Generator for NAMD, GROMACS, AMBER, OpenMM, and CHARMM/OpenMM Simulations Using the CHARMM36 Additive Force Field. *J. Chem. Theory Comput.* **2016,** *12* (1), 405-413.
8. Jorgensen, W. L.; Chandrasekhar, J.; Madura, J. D.; Impey, R. W.; Klein, M. L., Comparison of simple potential functions for simulating liquid water. *J. Chem. Phys.* **1983,** *79* (2), 926-935.
9. (a) Martyna, G. J.; Tobias, D. J.; Klein, M. L., Constant pressure molecular dynamics algorithms. *J. Chem. Phys.* **1994,** *101* (5), 4177-4189; (b) Feller, S. E.; Zhang, Y.; Pastor, R. W.; Brooks, B. R., Constant pressure molecular dynamics simulation: The Langevin piston method. *J. Chem. Phys.* **1995,** *103* (11), 4613-4621.
10. Essmann, U.; Perera, L.; Berkowitz, M. L.; Darden, T.; Lee, H.; Pedersen, L. G., A smooth particle mesh ewald method. *J. Chem. Phys.* **1995,** *103*, 8577–8592.
11. Pang, Y. T.; Miao, Y.; Wang, Y.; McCammon, J. A., Gaussian Accelerated Molecular Dynamics in NAMD. *J. Chem. Theory Comput.* **2017,** *13* (1), 9-19.
12. Feng, J.-J.; Chen, J.-N.; Kang, W.; Wu, Y.-D., Accurate Structure Prediction for Protein Loops Based on Molecular Dynamics Simulations with RSFF2C. *J. Chem. Theory Comput.* **2021,** *17* (7), 4614-4628.
13. Abraham, M. J.; Murtola, T.; Schulz, R.; Páll, S.; Smith, J. C.; Hess, B.; Lindahl, E., GROMACS: High performance molecular simulations through multi-level parallelism from laptops to supercomputers. *SoftwareX* **2015,** *1-2*, 19-25.
14. Bussi, G.; Donadio, D.; Parrinello, M., Canonical sampling through velocity rescaling. *J. Chem. Phys.* **2007,** *126* (1), 014101.
15. (a) Parrinello, M.; Rahman, A., Polymorphic transitions in single crystals: A new molecular dynamics method. *J. Appl. Phys.* **1981,** *52* (12), 7182-7190; (b) Nosé, S., A unified formulation of the constant temperature molecular dynamics methods. *J. Phys. Chem.* **1984,** *81* (1), 511-519.
16. Papadopoulos, J. S.; Agarwala, R., COBALT: constraint-based alignment tool for multiple protein sequences. *Bioinformatics* **2007,** *23* (9), 1073-9.

17. Franke, D.; Petoukhov, M. V.; Konarev, P. V.; Panjkovich, A.; Tuukkanen, A.; Mertens, H. D. T.; Kikhney, A. G.; Hajizadeh, N. R.; Franklin, J. M.; Jeffries, C. M.; Svergun, D. I., ATSAS 2.8: a comprehensive data analysis suite for small-angle scattering from macromolecular solutions. *J. Appl. Crystallogr.* **2017,** *50* (Pt 4), 1212-1225.
18. (a) Putnam, C. D.; Hammel, M.; Hura, G. L.; Tainer, J. A., X-ray solution scattering (SAXS) combined with crystallography and computation: defining accurate macromolecular structures, conformations and assemblies in solution. *Q. Rev. Biophys.* **2007,** *40* (3), 191-285; (b) Mertens, H. D.; Svergun, D. I., Structural characterization of proteins and complexes using small-angle X-ray solution scattering. *J Struct Biol* **2010,** *172* (1), 128-41.
19. Manalastas-Cantos, K.; Konarev, P. V.; Hajizadeh, N. R.; Kikhney, A. G.; Petoukhov, M. V.; Molodenskiy, D. S.; Panjkovich, A.; Mertens, H. D. T.; Gruzinov, A.; Borges, C.; Jeffries, C. M.; Svergun, D. I.; Franke, D., ATSAS 3.0: expanded functionality and new tools for small-angle scattering data analysis. *J. Appl. Crystallogr.* **2021,** *54* (Pt 1), 343-355.
20. Conroy, M. J.; Durand, A.; Lupo, D.; Li, X.-D.; Bullough, P. A.; Winkler, F. K.; Merrick, M., The crystal structure of the Escherichia coli AmtB-GlnK complex reveals how GlnK regulates the ammonia channel. *Proc. Natl. Acad. Sci. U.S.A.* **2007,** *104* (4), 1213-1218.
21. Campeotto, I.; Zhang, Y.; Mladenov, M. G.; Freemont, P. S.; A., G., Complex Structure and Biochemical Characterization of the Staphylococcus aureus Cyclic Diadenylate Monophosphate (c-di-AMP)-binding Protein PstA, the Founding Member of a New Signal Transduction Protein Family. *J. Biol. Chem.* **2015,** *290* (5), 2888-2901.
